# *Salmonella* in reptiles: a review of occurrence, interactions, shedding and risk factors for human infections

**DOI:** 10.3389/fcell.2023.1251036

**Published:** 2023-09-26

**Authors:** Michael Pees, Maria Brockmann, Natalie Steiner, Rachel E. Marschang

**Affiliations:** ^1^ Department of Small Mammal, Reptile and Avian Diseases, University of Veterinary Medicine Hannover, Hanover, Germany; ^2^ Laboklin GmbH and Co., KG, Bad Kissingen, Germany

**Keywords:** *Salmonella*, reptile, zoonosis, prevalence, interactions

## Abstract

*Salmonella* are considered a part of the normal reptile gut microbiota, but have also been associated with disease in reptiles. Reptile-associated salmonellosis (RAS) can pose a serious health threat to humans, especially children, and an estimated 6% of human sporadic salmonellosis cases have been attributed to direct or indirect contact with reptiles, although the exact number is not known. Two literature searches were conducted for this review. The first evaluated reports of the prevalence of *Salmonella* in the intestinal tracts of healthy reptiles. *Salmonella* were most commonly detected in snakes (56.0% overall), followed by lizards (36.9%) and tortoises (34.2%), with lower detection rates reported for turtles (18.6%) and crocodilians (9%). Reptiles in captivity were significantly more likely to shed *Salmonella* than those sampled in the wild. The majority of *Salmonella* strains described in reptiles belonged to subspecies I (70.3%), followed by subspecies IIIb (29.7%) and subspecies II (19.6%). The second literature search focused on reports of RAS, revealing that the highest number of cases was associated with contact with turtles (35.3%), followed by lizards (27.1%) and snakes (20.0%). Reptiles associated with RAS therefore did not directly reflect prevalence of *Salmonella* reported in healthy representatives of a given reptile group. Clinical symptoms associated with RAS predominantly involved the gastrointestinal tract, but also included fever, central nervous symptoms, problems with circulation, respiratory symptoms and others. Disease caused by *Salmonella* in reptiles appears to be dependent on additional factors, including stress, inadequate husbandry and hygiene, and other infectious agents. While it has been suggested that reptile serovars may cause more severe disease than human-derived strains, and some data is available on invasiveness of individual strains in cell culture, limited information is available on potential mechanisms influencing invasiveness and immune evasion in reptiles and in RAS. Strategies to mitigate the spread of *Salmonella* through reptiles and to reduce RAS focus mostly on education and hygiene, and have often been met with some success, but additional efforts are needed. Many aspects regarding *Salmonella* in reptiles remain poorly understood, including the mechanisms by which *Salmonella* persist in reptile hosts without causing disease.

## 1 Introduction

The role of *Salmonella* in reptiles and their zoonotic potential has been discussed for decades, and this discussion has, in some cases, been controversial. Reptile-associated salmonellosis (RAS) has been considered a threat to public health since the 1970’s, and a wide range of restrictions and measures have been suggested and in some cases implemented over the years in different countries around the world in order to mitigate the associated risk. A large number of scientific studies have focused on the detection of *Salmonella* strains in reptiles as well as their role in both reptile and human health.

*Salmonella* are often considered a normal part of the reptile gut microbiome (Pasmans et al., 2021). However, studies on the prevalence of these bacteria in specific reptile species and populations have led to widely varying numbers (e.g., Maciel et al., 2010; Scheelings et al., 2011). In addition, *Salmonella* have regularly been associated with disease in individual reptiles (e.g., Clancy et al., 2016b) as well as in disease outbreaks in groups of animals (e.g., Ramsay et al., 2002; Sakaguchi et al., 2017).

In general, the *Salmonella* detection rate in reptiles is highly dependent on the sample type, sampling technique, time of sampling, and sample quality, as well as on the technique used for detection, complicating the comparison of various studies (e.g., Marin et al., 2013; Fagre et al., 2020). A wide variety of methods, including pre-enrichment in various media and the use of a variety of agar types and incubation temperatures have been used for the detection of *Salmonella* in reptiles. Only few studies cite specific guidelines, such as the ISO 6579-1:2017 for the isolation of *Salmonella* from reptile samples (Zając et al., 2021; Merkevičienė et al., 2022). PCR is also frequently used for the detection of *Salmonella* in reptiles (e.g., Sánchez-Jiménez et al., 2011; Fagre et al., 2020; Marenzoni, 2022; Vorbach et al., 2022).

Studies focusing on describing the intestinal microbiome in various reptiles using molecular methods rather than culture have not found *Salmonella* to be a major part of the microbiome (e.g., Keenan and Elsey, 2015; Jiang et al., 2017; Ahasan et al., 2018; Shang et al., 2023) and in many cases, *Salmonella* has only been found in limited amounts under specific circumstances in studies evaluating the gut microbiome of specific species. For example, *Salmonella* were only detected in a single green turtle (*Chelonia mydas*) after rehabilitation, but not in any animals tested pre-hospitalization, leading authors to hypothesize that living under human care could be involved in colonization of the intestine with *Salmonella* and possibly spread in the environment (Ahasan et al., 2018). Similarly, Jiang et al. found that the presence of *Salmonella* in the gut microbiome of captive crocodile lizards (*Shinisaurus crocodilurus*) was dependent on what they were fed (Jiang et al., 2017). Generally, the microbiome in reptiles is reported to be sensitive to environmental factors, such as temperature, with small changes leading to shifting and destabilization (Bestion et al., 2017; Zhang, 2022).

There are reports documenting that some reptiles are colonized with *Salmonella* very early in life, possibly through the eggshell (Holgersson et al., 2016) as well as during pregnancy and birth (Schroter et al., 2006) indicating that *Salmonella* spp. can contribute to the physiological microbiome early in life. Other modes of transmission include prey or environmental contamination.

The risk of spreading *Salmonella* spp. from reptiles to humans (RAS), especially from pet reptiles to exposed children, has been known for decades. However, the main reptile species reported transmitting *Salmonella* spp. have changed over the years and may vary among different age groups of affected humans (De Jong et al., 2005; Sauteur et al., 2013). Transmission may also depend on the countries or continents evaluated, as different pet reptiles may be popular in different areas (Bertrand et al., 2008; Valdez, 2021). Originally, mostly small turtles such as *Trachemys* spp. (formerly *Pseudemys* spp.) were reported as risk factors and potential transmitters, especially in young children (Williams, 1965; Altman et al., 1972; Bradley et al., 2001). However, in the USA, this led to a ban on selling turtles with shell diameters smaller than 4 inches in 1975 to prevent children from putting them in their mouths (Hatt et al., 2009). This ban led to a drastic reduction in the number of human salmonellosis cases in the USA (Bradley et al., 2001). In the last decades, a shift towards lizards as the most likely transmitters for RAS has been noted (Böhme et al., 2009; Pees et al., 2013; Whitten et al., 2015; Whiley et al., 2017; Kiebler et al., 2020). In particular, bearded dragons (*Pogona* spp.) have been reported as a source of salmonellosis globally, including in Germany (Böhme et al., 2009; Haase et al., 2011; Weiss et al., 2011; Pees et al., 2013; Whiley et al., 2017), the United Kingdom (Cooke et al., 2009), the Netherlands (Berendes et al., 2007), Australia (Moffatt et al., 2010) and the USA (Lang et al., 2007; Tabarani et al., 2010; Lowther et al., 2011). However, other reptile species such as chameleons (Willis et al., 2002), geckos, anoles, snakes (Whitten et al., 2015; Krishnasamy et al., 2018) and iguanas (Nowinski and Albert, 2000; Cellucci et al., 2010) have also been reported as sources of human infection. Salmonellosis associated with turtles is also still reported regularly around the world (Harris et al., 2010; Basler et al., 2015; Kuroki et al., 2015; Walters et al., 2016; Lane et al., 2019; Lane et al., 2019; Okabe et al., 2021; Waltenburg et al., 2022).

RAS is believed to account for at least 6% of all human salmonellosis cases, depending on region and age of the affected patients (Bradley et al., 2001; Wells et al., 2004; Meletiadis et al., 2022). It is usually a problem of children rather than adults (Ackman et al., 1995; Willis et al., 2002; Harris et al., 2010; Weiss et al., 2011; Lafuente et al., 2013; Whitten et al., 2015). Some studies also indicate that RAS occurs more often in younger children than salmonellosis of other origins and may also be associated with an increased hospitalization rate (Colomb-Cotinat et al., 2014; Sauteur et al., 2013; Murphy and Oshin, 2015). Especially young children are prone to severe manifestations with dehydration, sepsis, and meningitis which may be life-threatening (Hatt et al., 2009; Haase et al., 2011). One study also found younger children to be more prone to severe disease resulting in hospitalization (Cellucci et al., 2010). However, there are also multiple case reports describing RAS in adults (Stam et al., 2003; Brenneman et al., 2022; Castlemain and Castlemain, 2022) and a few years ago, a shift towards RAS cases in adults was reported (Mughini-Gras et al., 2016). Comorbidities are frequently reported in adults that develop bacteriaemia (Lane et al., 2019; Barry and Finn, 2022; Brenneman et al., 2022). Common clinical signs of RAS include gastrointestinal symptoms such as (bloody) diarrhea, vomitus, abdominal cramping as well as fever (Willis et al., 2002; Böhme et al., 2009; Harris et al., 2010; Weiss et al., 2011; Colomb-Cotinat et al., 2014). As mentioned above, invasive cases have been described and can encompass septicemia, meningitis, and bone or joint infection (Nowinski and Albert, 2000; Lang et al., 2007; Van Meervenne et al., 2009; Colomb-Cotinat et al., 2014; Sauteur et al., 2013; Murphy and Oshin, 2015).

This review aimed to collect end evaluate published data on *Salmonella* in reptiles, with a focus on their detection, interactions with their hosts, and relevance as a zoonotic pathogen. Special emphasis was placed on scientific papers published within the last 2 decades to provide a comprehensive overview and interpretation of the state of research, but also to determine gaps in our understanding of these bacteria in reptile hosts and necessary research steps for the future.

## 2 *Salmonella* as pathogens in reptiles

Despite the frequent detection of *Salmonella* in healthy reptiles and their possible role in the normal reptile microbiome, these bacteria have also been associated with disease in many cases. Disease outbreaks affecting multiple animals within a collection have also been described (Sakaguchi et al., 2017). However, the role of *Salmonella* as primary pathogens in reptiles is not always clear. Evaluation of their role in disease processes can be challenging. Several possible predisposing factors have been reported for the development of clinical salmonellosis in reptiles. These include stress (Clancy et al., 2016a; Bertolini et al., 2021), parasite infestations (Sting et al., 2013), viral disease (Sting et al., 2013), metabolic disease (Sting et al., 2013), and inappropriate husbandry (Bertolini et al., 2021). Snakes have been reported to have a higher risk of salmonellosis, followed by lizards, and crocodilians, with the lowest risk of disease reported for chelonians (Grupka et al., 2006; Clancy et al., 2016a). Snakes seem especially prone to clinical diseases caused by *Salmonella* ssp. IIIa and IIIb (Grupka et al., 2006). Clinical signs of a *Salmonella*-associated disease in reptiles are highly variable and often non-specific. Septicaemia (Orós et al., 1996; Mitchell and Shane, 2001; Téllez et al., 2002; Grupka et al., 2006; Köbölkuti et al., 2013) pneumonia (Orós et al., 1996; Mitchell and Shane, 2001; Schroff et al., 2010; Sakaguchi et al., 2017), enterocolitis (Sakaguchi et al., 2017), abscesses (Barboza et al., 2018; Hyeon et al., 2021), osteomyelitis, especially in snakes (Isaza et al., 2000; Ramsay et al., 2002; Grupka et al., 2006; Di Girolamo et al., 2014; Clancy et al., 2016b; Bertolini et al., 2021), dermatitis (Clancy et al., 2016a; Sakaguchi et al., 2017; Bertolini et al., 2021), splenitis (Sakaguchi et al., 2017; Bertolini et al., 2021), and hepatitis (Bertolini et al., 2021) have all been described in multiple cases. Clinical signs observed can include anorexia, lethargy, cachexia, paresis, dyspnoea, hypovolemic shock, and sudden death (Ramsay et al., 2002; Jacobson, 2007; Bölcskei et al., 2009; Gorman, 2010; Mayer and Donnelly, 2012; Silva-Hidalgo et al., 2014; Clancy et al., 2016a).

Pathological and histological changes associated with cases of reported salmonellosis include necrotizing inflammation in various tissues including e.g., the intestine, spleen, and lung in affected crocodilians (Sakaguchi et al., 2017), and renal granulomas in sea turtles (Work et al., 2019). Necrotizing and granulomatous inflammation have also characterized lesions associated with *Salmonella* in a wide variety of tissues in various snake species including the gastro-intestinal tract, liver, spleen, blood vessels, and myocardium (Pasmans et al., 2021). Snakes with osteomyelitis associated with *Salmonella* infections have been found to have diffuse heterophilic-granulomatous osteomyelitis and discrete heterophilic granulomas (Ramsay et al., 2002).

## 3 Systematic review

### 3.1 *Salmonella* detection in healthy reptiles

To evaluate the occurrence of *Salmonella* spp. in healthy reptiles throughout the world and among specific animal groups, a systematic literature review was conducted (modified PRISMA flow diagram see Supplementary Data Sheet S1) including the Pubmed query: [(*salmonella*) AND (reptile) AND (prevalence)] AND {[“2003/01/01” (Date—Publication): “3,000” (Date—Publication)]}. On 14 April 2023, this resulted in a total of 219 results published between 2003 and 2023. Inclusion criteria for further evaluation were: at least 10 individual animals tested, sample location (e.g., cloaca) provided, no clinical signs of disease reported, at least one enrichment procedure conducted, and identification of isolates at least to subspecies level. Reviews were excluded from further evaluation. This resulted in 77 studies that were further evaluated. A summary of the obtained data is given in Tables 1, 2. Data points used for the evaluation and reference to all studies included are provided in Supplementary Data Sheet S2. For the prevalence results, statistical analysis for significant differences was performed using the commercial software SPSS 28.0 (IBM, Armonck, United States) and the Mann-Whitney-U-Test. A significance was assumed with *p* ≤ 0.05.

**TABLE 1:** Literature review of prevalence of *Salmonella* in healthy reptiles, number of studies and total number of animals included (grey) (reptile nonspec. = data could not be assigned to a specific reptile group). Data points used for the evaluation and references to all studies included are provided in Supplementary Data Sheet S2.

	data sets	All	Captive	Wild	Europe	North America	South America	Africa	Asia	Oceania
Publications		77	39	38	26	15	10	2	14	10
Data sets		138	77	53	51	18	14	2	24	29
Reptiles total	138	12,557	4,766	6,818	3,236	1765	853	102	3,799	2,802
Snakes	34	1868	1,379	452	1,020	197	37	0	402	212
Lizards	42	6,198	1,091	4,224	672	875	527	74	2,598	1,452
Tortoises	16	819	642	177	562	80	10	28	4	135
Turtles	32	2,611	1,278	1,301	739	596	142	0	795	339
Crocodilians	5	159	105	33	2	0	123	0	0	34
Tuatara	3	630	30	600	0	0	0	0	0	630
Reptile non spec	6	272	241	31	241	17	14	0	0	0

**TABLE 2:** Literature review of prevalence of *Salmonella* in healthy reptiles, reported *Salmonella* prevalences [% of animals].

Prevalence rates	All animals	Captive	Wild
Average	34.5	42.9	24.3
Maximum rate	100.0	100.0	100.0
Minimum rate	0.0	0.0	0.0
Snakes	56.0	68.1	31.7
Lizards	36.9	52.0	26.3
Tortoises	34.2	31.8	39.6
Turtles	18.6	19.2	17.9
Crocodilians	9.0	9.2	3.0
Tuatara	0.0	0.0	0.0

Altogether, results from 12,557 individual reptiles were included. Prevalence studies were conducted on all continents except Antarctica, and many studies included reptiles from different groups or studies from both captive and wild animals. The data were evaluated for six different reptile groups (snakes, lizards, tortoises, turtles, crocodilians, tuatara) corresponding in part to taxonomic order (crocodilians, tuatara) or family (tortoises) and in part to lifestyle (turtle) or recognized groups within a joint order (snakes and lizards), as well as source (wild *versus* captive, or unclear). The data contained in the 77 publications were divided into 138 data sets: each for a single reptile group and source. These sets were the basis for all evaluations and calculations made here (Table 1). The methods used for species and serovar identification in the survey conducted here were also compared. Of all data evaluated, characterization of the *Salmonella* isolates was based on serological methods in 111 data sets. Molecular methods were used for characterization in 27 data sets. In 14 of these 27, both molecular and serological methods were used. The method used for identification was not clearly described in 14 data sets, either because no *Salmonella* were detected in the respective study, or due to reference to an external laboratory without explaining the method. Altogether, 86 data sets reported complete serovar identification, only a subset of the isolates were further characterized in 15 datasets, and identification was only reported to the subspecies level in 13 datasets. Details on the methods used in individual studies are reported in Supplementary Data Sheet S2. Statistical evaluation was only conducted for the reported prevalence depending on the source and the reptile group (crocodilians and tuataras were excluded due to the low number of data sets). A statistical evaluation of other results was deemed unreliable due to differences in the study designs.

Most frequently, reptile samples examined for *Salmonella* spp. are of cloacal or fecal origin, but studies on detection in tissue or environmental samples are also available. In our literature review, 73.9% of the samples analysed were cloacal swabs, while 37.7% were feces, and 15.9% were other types of samples. Comparing different studies regarding the time between collection and arrival of the samples in the laboratory, these ranged from less than 24 h (e.g., Corrente et al., 2017; Bjelland et al., 2020; Baling and Mitchell, 2021; Merkevičienė et al., 2022; Song et al., 2023) up to 72 h (e.g., Zając et al., 2021).

**FIGURE 1:**
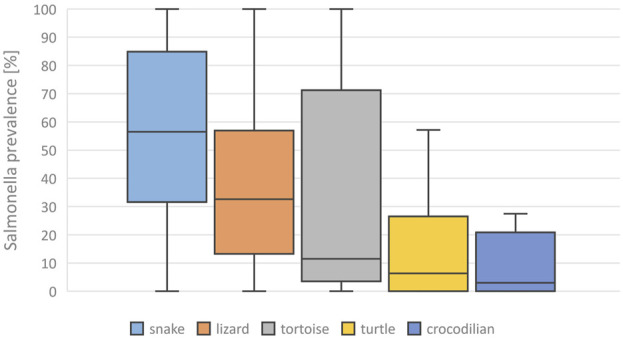
Evaluation of reported detection rates of *Salmonella* in healthy reptiles, from literature published between 2003 and 2023, based on 138 data sets (for each reptile group and source) extracted from 77 individual publications: *Salmonella* prevalences [%] reported for different reptile groups, Boxplots (median, 1. and 3. quartile, maximum reported detection rate).

**FIGURE 2:**
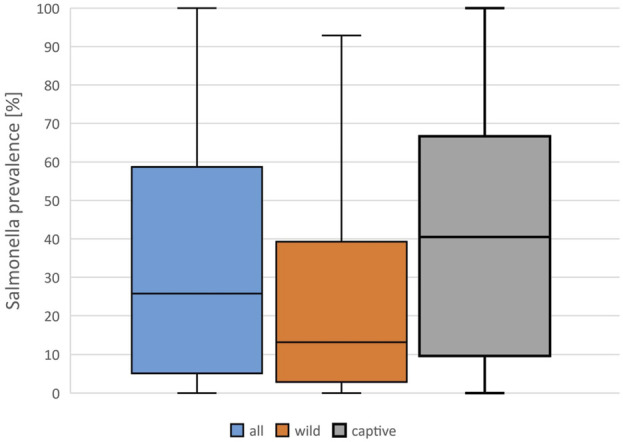
Evaluation of reported detection rates of *Salmonella* in healthy reptiles, from literature published between 2003 and 2023, based on 138 data sets (for each reptile group and source) extracted from 77 individual publications: *Salmonella* prevalences [%] reported in all data sets, and results for captive and wild reptiles, Boxplots (median, 1. and 3. quartile, maximum reported detection rate).

A mean *Salmonella* prevalence of 34.5% was calculated overall for all groups and studies. The prevalence reported in the studies varied between the reptile groups, with the highest rate of 56.0% in snakes, lower in turtles (18.6%) and the lowest in crocodilians (9.0%) and tuatara (0.0%), the latter two based on a limited number of data sets (Table 2; Figure 1). The differences in the reported prevalence were significant between snakes and lizards (*p* = 0.025), tortoises (*p* = 0.047), and turtles (*p* ≤ 0.001). *Salmonella* detection rates were also significantly lower in turtles than in lizards (*p* ≤ 0.001). Regarding the source of the reptile, a significantly (*p* = 0.002) higher mean *Salmonella* prevalence was noted for those reptiles living in captivity than for those sampled in the wild (Figure 2). A higher prevalence in captive animals could also be confirmed within the reptile groups except for tortoises, being significant for the group of snakes (*p* = 0.002) and lizards (*p* = 0.002). In general, the range of the reported prevalence was very broad and ranged between zero or close to zero and 100%. This broad range was found for all reptiles as well as for each group and source separately.

**FIGURE 3:**
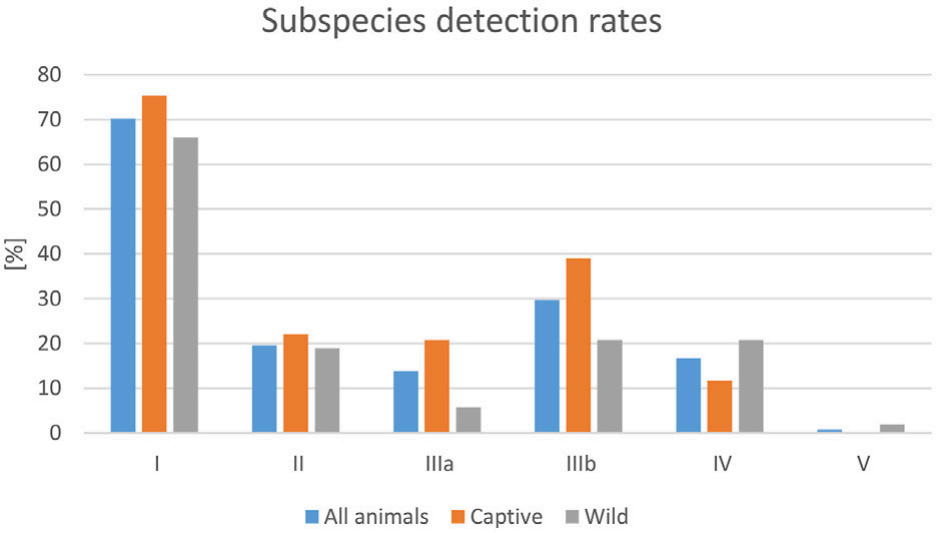
Evaluation of reported detection rates of *Salmonella* in healthy reptiles, from literature published between 2003 and 2023, based on 138 data sets (for each reptile group and source) extracted from 77 individual publications: frequencies of *Salmonella* subspecies reported [%].

Besides the detection rate, the detected serovars also varied greatly between the different studies. By far the most frequently identified serovars in the analyzed studies belonged to subspecies I (70.3% reported rate per data sets), followed by subspecies IIIb (29.7%), and subspecies II (19.6%) (Figure 3). This dominance of subspecies I serovars was also seen for both captive and wild reptiles, although the detection rates for serovars from individual subspecies differed between wild and captive reptiles, and *Salmonella* ssp. V isolates were only reported from wild reptiles. For subspecies I, a broad range of serovars were reported in the evaluated publications, with the following serovars named most often (this list is not exhaustive): Abony, Amsterdam, Eastbourne, Fluntern, Halle, Heron, Java, Javiana, Kottbus, Newport, Oranienburg, Pomona, Poona, Potsdam, Rubislaw, Weltevreden.

### 3.2 Reptile-human transmission

**FIGURE 4:**
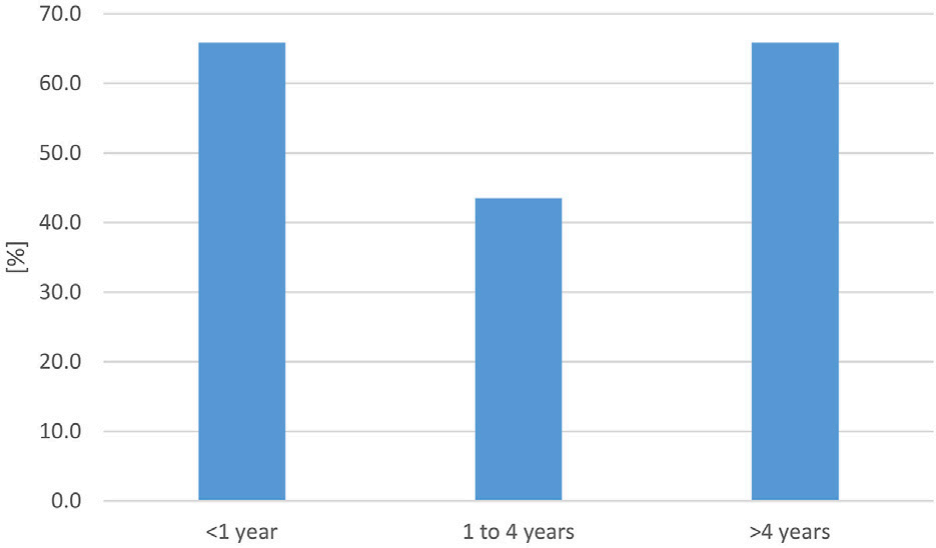
Evaluation of reported reptile-associated salmonellosis (RAS), from literature published between 2003 and 2023, based on 79 data sets (for each reptile group and source) extracted from 53 individual publications: percentage [%] of data sets that reported the respective age group to be affected by reptile-associated salmonellosis (RAS). Many reports included information on several affected age groups.

To evaluate reports on reptile-associated salmonellosis (RAS) throughout the world with regard to occurrence, symptoms, and reptile species involved, a systematic literature review was conducted [modified PRISMA flow diagram see Supplementary Data Sheet S3) using the formula: Pubmed query: {[(*salmonella*) AND (reptile) AND [(human) or (zoonosis) or (RAS)]} AND {[“2003/01/01” (Date—Publication): “3,000” (Date—Publication)]} NOT (review)]. On 17 May 2023, this resulted in a total of 264 results published between 2003 and 2023. Inclusion criteria were a clear association of *Salmonella* spp. infection in humans and reptile contact, further classification of reptile contact and not just mentioned as a possible risk factor, no review article as well as text written in English, French, or German, resulting in 53 studies that were further evaluated. A summary of the obtained data is given in Table 3. Data points used for the evaluation and reference to all studies included are given in Supplementary Data Sheet S4. As for the prevalence survey, data was evaluated for six different reptile groups (snakes, lizards, tortoises, turtles, crocodilians, tuatara), as well as the source of the reptile (wild, pet, unknown). The data contained in the 53 publications were divided into 85 data sets: each for a single reptile group and source. Overall, 3,025 human cases were described. Studies were conducted mainly in Europe (47) and North America (32), and a few studies in Asia (3), Oceania (2), and Africa (1). The data was evaluated for five different reptile groups [snakes, lizards, tortoises, turtles, and reptile non-specific (for cases in which the information provided in the publication did not allow a clear identification of the reptile group involved)]; no infections associated with crocodilians or tuataras were reported in any of the evaluated studies. In 79 data sets, identification of the *Salmonella* isolates to the serovar level was confirmed, whereas in 6 studies, identification was incomplete. Most studies used molecular methods, including pulsed field gel electrophoresis (42) for isolate characterization, 34 used serologic methods (10 additionally to molecular methods). In 24 studies, identification methods were inconsistent or not clearly described (details in Supplementary Data Sheet S4). Reports on *Salmonella* subspecies and animal groups involved were only compared statistically between Europe and North America, as the number of studies on other continents was limited (SPSS 28.0 (IBM, Armonck, United States); Mann-Whitney-U-Test, significance assumed with *p* ≤ 0.05). As RAS is reported to affect especially young children, we calculated three age groups (less than 1 year, 1–4 years, and older than 4 years). The age reporting rates detected are shown in Figure 4.

**TABLE 3:** Literature review of reptile-associated salmonellosis (RAS): Number of data sets, total number of humans involved, and frequency of reptile groups reported to be involved [% of data sets] (reptile nonspec. = reptile data could not be assigned to a specific reptile group). Data points used for the evaluation and references to all studies included are provided in Supplementary Data Sheet S4.

		All	Europe	North Am	Africa	Asia	Oceania
No. of publications		53	24	23	1	3	2
No. of data sets		85	47	32	1	3	2
No. of affected humans		3,025	204	2,805	1	7	8
Reptile group involved	snakes %	20.0	27.7	9.4	0.0	33.3	0.0
	lizards %	27.1	21.3	34.4	100.0	0.0	50.0
	tortoises %	3.5	4.3	0.0	0.0	33.3	0.0
	turtles %	35.3	31.9	40.6	0.0	33.3	50.0
	reptile nonspec. %	11.8	10.6	15.6	0.0	0.0	0.0

**FIGURE 5:**
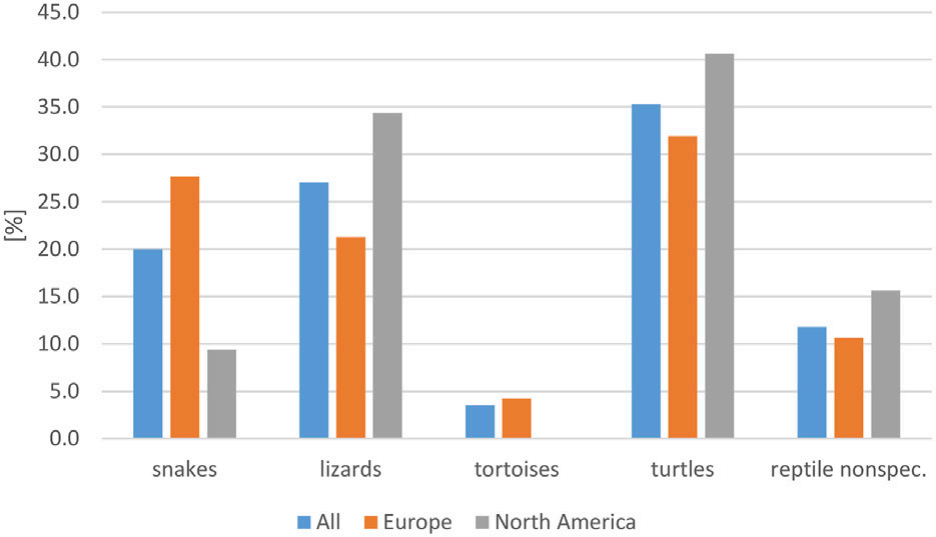
Evaluation of reported reptile-associated salmonellosis (RAS), from literature published between 2003 and 2023, based on 79 data sets (for each reptile group and source) extracted from 53 individual publications: frequency of reptile groups reported as the source of reptile-associated salmonellosis (RAS), for all data sets evaluated and for Europe and North America (reptile nonspec. = reptile data could not be assigned to a specific reptile group).

The majority of the reports included turtles (35.3%), lizards (27.1%), and snakes (20%), whereas only two studies in Europe and one study in Asia (3.5%) mentioned tortoises (Table 3). The reptile groups most frequently involved in RAS differed between Europe and North America (Figure 5), although the highest number of cases was reported to involve turtles on both continents. The difference in frequency of an association with contact with snakes was significant between these two continents (*p* = 0.035). The specific reptile species involved in RAS cases were often not provided. For those cases in which species was provided, the species mentioned most often were bearded dragons (*Pogona vitticeps*, 14 studies), followed by sliders (*Trachema script*a spp., seven studies), green iguanas (*Iguana iguana*, six studies) and Chinese water dragons (*Physignathus coincinus*, five studies).

**FIGURE 6:**
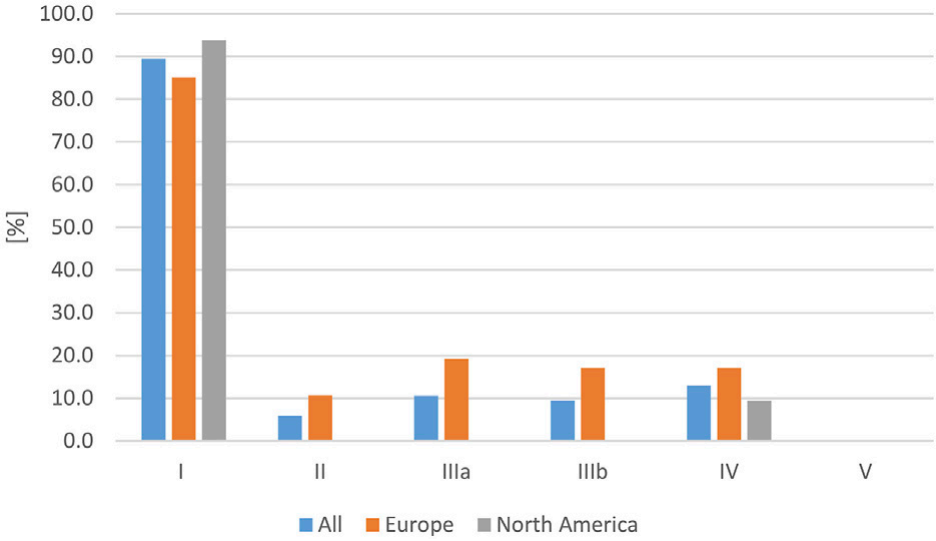
Evaluation of reported reptile-associated salmonellosis (RAS), from literature published between 2003 and 2023, based on 79 data sets (for each reptile group and source) extracted from 53 individual publications: frequency of *Salmonella* subspecies reported as a cause of reptile-associated salmonellosis (RAS) in all studies as well as only in Europe and only in North America in % of data sets in which each subspecies was reported [%].

Although most studies on RAS were published in Europe, the number of individually affected humans was much higher in North America, due to the higher number of reported individuals per study. With respect to the isolated serovar in human cases, in 49.4% of all studies, the respective serovar was confirmed in a reptile. In 89.4% of all studies, a *Salmonella* subspecies I serovar was isolated, followed by a detection rate of 12.9% of subspecies IV and 10.6% of subspecies IIIa. The isolation rates are shown in Figure 6 for North America and Europe. Subspecies IIIa and IIIb were found to be significantly more often reported in RAS cases in Europe than in North America (*p* = 0.019 and *p* = 0.012). The subspecies I serovars most often reported included (this list is not exhaustive): Pomona (13/53), Poona (8/53), Apapa (5/53), Monschaui (4/53), Muenich (4/53), Oranienburg (4/53), Cotham (3/53), and Newport (3/53).

Transmission of *Salmonella* between reptiles and humans can occur due to direct or indirect contact with infectious reptile-associated material. Indirect contact leading to RAS is frequently reported and may include living in the same home (Tabarani et al., 2010; Weiss et al., 2011; Colomb-Cotinat et al., 2014), contact with infected feeder mice (Harker et al., 2011; Cartwright et al., 2016; Kanagarajah et al., 2018; Marin et al., 2018; Vrbova et al., 2018), or contaminated surfaces, e.g., of reptile enclosures (Friedman et al., 1998; Hatt et al., 2009). This has even been described several weeks after the removal of the reptile (Weiss et al., 2011). Spread of RAS throughout the home is also possible, as reptile-associated *Salmonella* has even been found in vacuum cleaners (Whiley et al., 2017). Humans may also serve as vectors, e.g., an adult working in a pet reptile shop transmitting *Salmonella* to a child at home or a reptile owner transmitting *Salmonella* during a visit to another home (Weiss et al., 2011). In our literature review, about one-quarter of the publications (25.9%) included details on a direct infection, and one-quarter on indirect contamination (27.1%).

**FIGURE 7:**
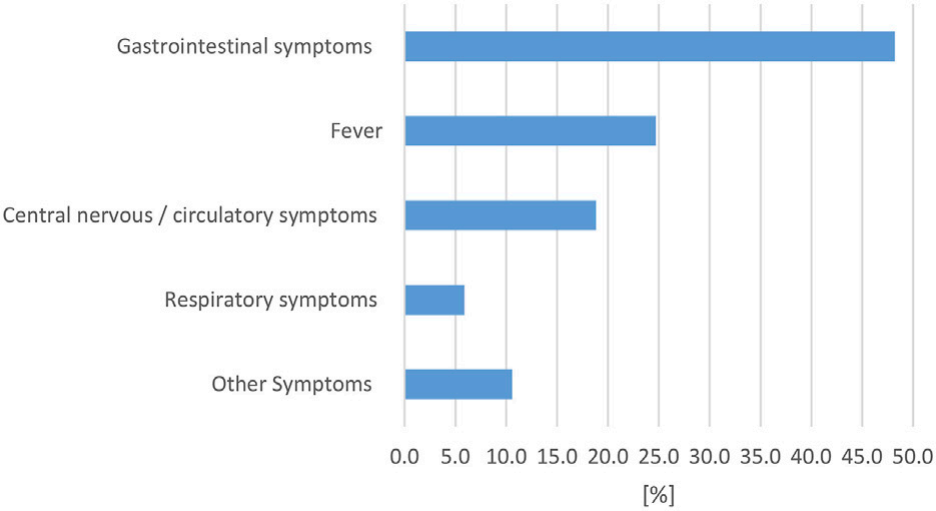
Evaluation of reported reptile-associated salmonellosis (RAS), from literature published between 2003 and 2023, based on 79 data sets (for each reptile group and source) extracted from 53 individual publications: percentage [%] of data sets that reported the respective clinical symptom complex in human cases with reptile-associated salmonellosis (RAS).

Described symptoms in affected humans were classified in the following categories: gastrointestinal symptoms (including diarrhea, vomiting, abdominal cramps, nausea), fever, respiratory symptoms, central nervous and/or circulatory symptoms (including meningitis, signs of sepsis, shock), and other symptoms. Symptoms in multiple categories were possible and gastrointestinal symptoms were most commonly reported (Figure 7).

## 4 Discussion

Despite a plethora of publications and studies documenting *Salmonella* carriage in reptiles, there are still many open questions regarding the interaction between *Salmonella*, reptiles, the environment, the reptile immune system, and disease, as well as the factors influencing transmission between reptiles and humans and options for mitigating the risks associated with *Salmonella* in reptiles. These are questions that are of interest for human and veterinary medicine as well as for environmental health and conservation.

### 4.1 *Salmonella* in reptiles

The overview of recent (in the past 20 years) studies on the detection of *Salmonella* in healthy reptiles conducted for this review supports a wide range of previous work indicating both that these bacteria are common in the intestinal tract of reptiles and that their distribution can vary widely under various circumstances and in various environments and reptile species. Variations in samples and methods employed for *Salmonella* spp. detection are also known to influence detection rates and methods used for *Salmonella* detection in reptiles can vary widely.

The differences in *Salmonella* prevalence found in this literature search are similar to those reported previously in a number of individual studies. Specifically, lower detections rates in chelonians in comparison to snakes or lizards have been repeatedly reported (Geue and Löschner, 2002; Scheelings et al., 2011; Hydeskov et al., 2013; Lukac et al., 2015; Corrente et al., 2017; Bjelland et al., 2020; Cota et al., 2021; Zając et al., 2021). However, other studies have found a higher prevalence in chelonians (Ebani et al., 2005; Bertelloni et al., 2016). Differences in prevalence between wild-caught animals and captive collections have also been reported, with generally lower detection rates in wild animals (Geue and Löschner, 2002; Scheelings et al., 2011).

A lack of detection of *Salmonella* in a sample should always be considered as a snap shot, with limited value for a permanent assessment. Reptiles that carry *Salmonella* in their gastro-intestinal tracts have repeatedly been shown to shed these bacteria irregularly (Maciel et al., 2010; Goupil et al., 2012; McWhorter et al., 2021), and shedding is known to be influenced by a variety of factors including husbandry conditions (climate, hygiene, diet). For example, carnivores have been shown to be more prone to increased shedding, especially if rodents are fed (Scheelings et al., 2011). Stress has been shown to increase shedding in various reptiles (DuPonte et al., 1978; Smith et al., 2012; Marin et al., 2013). Beside these variations, almost all studies evaluated focused on the qualitative detection of *Salmonella*, but—in contrast to the food industry—not on a quantitative assessment of the number of bacteria shed by reptiles. Our findings indicate that shedding of *Salmonella* is influenced by both the reptile group and the reptile habitat, which can have implications for zoonotic risk assessment. Among the reptile groups, snakes were most often *Salmonella* positive, with significantly higher detection rates found in this group than in all other reptile groups evaluated. Interestingly, the shedding frequency for individual reptile groups did not correlate with their apparent importance in RAS–we address this discrepancy below.

The physiological role of *Salmonella* in reptiles is still unclear. Saprophytic relationships have been suggested (Jacobson, 2007; Baling and Mitchell, 2021). The pathogenesis of salmonellosis in reptiles is still not understood, but there are several extrinsic and intrinsic factors that are discussed to have a significance influence on the colonization as well as disease development in reptiles:

Following colonization of the gut, translocation across the gut wall and hematogenous spread throughout the body can occur and can lead to systemic disease (Wellehan and Gunkel, 2004). Pasmans et al. (2003) demonstrated the role of the reptile intestinal mucosal layer as the site for *Salmonella* colonization, but also as a protection against invasion of the underlying tissue structures and systemic spread. Detection in inner organs with no detectable inflammation or clinical signs has also been reported following oral administration of *Salmonella* isolates in healthy bearded dragons (*P. vitticeps*) (Pasmans et al., 2013; Renfert et al., 2021). In addition to the role of the intestinal barrier, other tissues have also been hypothesized to be possible entry ways for systemic salmonellosis. Lung tissue was found to be a possible entry site for *Salmonella* spp. infections, especially after initial viral damage to the epithelium. In snakes, *Salmonella* have been reported to be part of the oral cavity microbiota, but not a normal finding in the lower airways (Plenz et al., 2015). However, after experimental application of a ferlavirus (a virus targeting the lung epithelium cells) in corn snakes (*Pantherophis guttatus*), *Salmonella* spp. was found to be the dominant bacterium causing pneumonia (Pees et al., 2019). A further study demonstrated a massive immune response to this secondary infection including interstitial infiltration of heterophils and mononuclear cells (Starck et al., 2017). A case report on a Burmese python (*Python molurus*) suffering from pneumonia with subsequent development of *endocarditis valvularis* involving *Salmonella* indicates that lung infections may lead to hematogenous dissemination of *Salmonella* to internal organs (Schroff et al., 2010).

Several studies (Pasmans et al., 2002; Clancy et al., 2016a; Bertolini et al., 2021) have suggested that the disparity in shedding and clinical signs observed in *Salmonella* spp. infection between mammals and reptiles may be attributed to differences in their immune systems. In mice, the entry of *Salmonella* spp. into the intestinal wall through Peyer’s patches and M-like cells has been extensively documented. However, the presence of Peyer’s patches has not been observed in reptilian species, although M-cell-like structures have been identified in the esophagus of adult turtles (Pasmans et al., 2002). This absence of Peyer’s patches and M-like cells in reptiles could explain why *Salmonella* typically does not invade the intestinal wall in reptiles that carry these bacteria in their guts.

Temperature likely also influences the replication and distribution of *Salmonella* in ectothermic reptiles. Pasmans et al., 2002 demonstrated that intestinal infection with *Salmonella* Muenchen can occur in a turtle (*Trachemy scripta* ssp. *scripta*) when kept at a temperature of 37°C, which is similar to the core temperature of mammals. The ability of *Salmonella* to invade intestinal tissue and colonize internal organs at temperatures typical of homeothermic animals may be attributed to several factors. Firstly, higher temperatures promote increased bacterial growth and higher shedding numbers due to enhanced doubling rates. Additionally, elevated temperatures could disrupt the normal functioning of the intestinal barrier, leading to invasion (Pasmans et al., 2002). Temperature has also been hypothesized to influence intra-intestinal biofilm formation and regulation of *Salmonella* spp. in reptiles (Renfert et al., 2021). These poikilothermic animals, with their dependence on external sources of heat for temperature regulation and diurnal changes in body temperature, may exhibit differences in biofilm formation compared to homeothermic organisms, as no biofilm formation was reported at 37°C, but was found below specific temperature thresholds (30°C) (Gerstel and Romling, 2001; Speranza et al., 2011).

Differences in virulence factors of specific *Salmonella* serovars at different temperatures may contribute to increased invasiveness (Pasmans et al., 2002). The influence of body temperature on the induction of *Salmonella* pathogenicity islands I and HilA genes, which play a major role in the intestinal phase of infection in homeotherms, remains unknown. Individual reports have indicated that specific serovars may be more pathogenic for some reptiles than others (Bemis et al., 2007), but this has not been sufficiently studied to differentiate between effects specific to a bacterial strain and effects of host species, immune status, and environment.

### 4.2 Reptile-human transmission of *Salmonella*

The second survey conducted in this review aimed to provide an overview of recent (from the past 2 decades) reports on human salmonellosis associated with reptiles, RAS. Again, it is important to acknowledge the limitations of this survey, including the same constraints mentioned for the prevalence review. Another restriction is the limitation of published studies available almost exclusively to Europe and North America. This may be due to differences in attitudes toward publication of such cases. We consider it unlikely that the bias in numbers of publications represents a difference in inherent risk of RAS in different parts of the world, although cultural differences in keeping and treatment of pet reptiles could play a role.

A consistent finding across the reports was that turtles were the reptile group most frequently named as a source of RAS, followed by lizards and, notably, snakes. The involvement of lizards and snakes varied, with lizards more often associated with RAS in North America. Interestingly, tortoises did not play a significant role in the reports, being identified as a source in only 3.5% of the cases. This is noteworthy considering the popularity of tortoises as pets, and the finding in the review of *Salmonella* prevalence that they frequently carry *Salmonella* (35% compared to 18% in turtles). Several factors likely contribute to these discrepancies:

Firstly, the study data do not directly reflect the popularity of specific reptile groups as pets, and therefore the possible exposure to humans. It is difficult to obtain reliable data on popularities of reptile species as pets. However, a study using google trends for analysis of changes in the reptile pet trade found that bearded dragons are currently by far the most popular reptiles traded, especially in the USA and Europe, whereas red-eared sliders (a common turtle species) have declined in popularity (Valdez, 2021). This is relevant for the association between bearded dragons and RAS, since this was the species most often identified in recent reports on RAS. The relevance of possible changes in the trade of turtles cannot be estimated at this point.

Secondly, the level of direct contact between reptiles and owners varies depending on the reptile species as well as on the owner. It is possible that the probability of close contact between owners and pet reptiles and especially between children and pet reptiles may vary depending on the reptile species, with lower average levels of contact with snakes than turtles and lizards.

Thirdly, for *Salmonella* transmission, the direct environment may play an important role, and most turtle species are kept in water, which may well serve as a vector (wet hands, wet material). Water—especially if hygiene measures are poor—can be contaminated with reptile-derived *Salmonella* (Mermin et al., 2004; Corrente et al., 2017; Waltenburg et al., 2022).

The differences noted in detection rates between wild reptiles and reptiles in captivity could—as mentioned above—also be due to differences in conditions, with dietary, stress and crowding-related factors likely causing the higher detection rates found in reptiles in captivity. Given the high dependency of ectothermic organisms on their environment, and natural living situations probably reflecting more physiological conditions than those in captivity, this could contribute to a stronger immune system and reduced stress (DuPonte et al., 1978; Geue and Löschner, 2002; Dickinson et al., 2005; Scheelings et al., 2011; Cota et al., 2021).

In general, the most important source of salmonellosis in humans is contaminated food (Bradley et al., 2001; Faulder et al., 2017; Meletiadis et al., 2022). Contaminated food can play a role in RAS, but direct consumption of *Salmonella*-contaminated reptile meat leading to human infection has also been described. Marine turtles (O’Grady and Krause, 1999), lizards (Ríos et al., 2022), terrapins, and crocodilians (Madsen, 1996; Magnino et al., 2009; López-Quintana et al., 2015; Draper et al., 2017) are all used as a source of food and their meat can be a source of salmonellosis, as can food contaminated with excretions of reptiles. The latter was reported for example, after a Thanksgiving dinner prepared by a bearded dragon owner (Lowther et al., 2011).

Age may also influence the risk for RAS. A study investigating the development of RAS in children concluded that the age of the children and the reptile group are significant risk factors, with children at a median age of 0.17 years affected more than the group with a median age of 2.0 years, and turtles being significantly more involved than other reptile groups (Sauteur et al., 2013). Another recent study, however, reports a shift of RAS cases to adulthood, while cases in children decreased (Mughini-Gras et al., 2016). Those and other studies (Pees et al., 2013) also conclude that hygienic insufficiencies are relevant risk factors when keeping reptiles.

Our literature survey confirmed the importance of subspecies I strains (almost 90%) for zoonotic risk. The prevalence study also confirmed subspecies I as the predominant *Salmonella* subspecies found in the reptile gastrointestinal tract, but to a lesser degree (about 70%). The zoonotic potential of reptile-derived *Salmonella* strains was examined on a cellular basis using Caco-2 cells to test for invasiveness. Pasmans et al. (Pasmans et al., 2005) found that saurian isolates belonging to subspecies I invaded human colon cells, Caco-2-cells, to a higher extent than isolates belonging to other subspecies, and to a higher extent than corresponding human serotypes. This was also confirmed by a more recent study on pet reptiles in Beijing, in which more than 50% of the *Salmonella* isolated were found to be more cytotoxic to Caco-2-cells than a defined standard strain (*Salmonella* Typhimurium 1,344) (Song et al., 2023). Additionally, those authors found that highly virulent strains most often belong to subspecies I and IIIb, and lizards seemed to have the highest prevalence with higher cytotoxicity followed by turtles and snakes. Among the tested isolates, strain 1101PV5, belonging to the serovar Pomona, showed the highest pathogenicity on the Caco-2-cells. McWorhter et al. (2021) tested the invasiveness of different *Salmonella* strains isolated from both captive and wild reptiles in human Caco-2 and mouse macrophage (J774A.1) cell lines, yielding diverse results. All isolates demonstrated invasiveness in both cell lines, with subspecies I and IV strains exhibiting the highest degree of invasiveness, while subspecies IIIa and IIIb strains displayed the lowest. The assessment of invasiveness in macrophages was undertaken due to its importance in within-host survival and replication. All isolates exhibited lower invasiveness in J774.1A macrophages compared to Caco-2 cells. The presence of virulence genes in isolates from reptiles was also examined by PCR (Pasmans et al., 2005), and no significant differences were found in the distribution of the virulence genes agfA, shdA, sopE, pefA, and spvR between subspecies I serovars from reptiles and the corresponding serovars from humans. Also, the presence of pathogenicity islands with virulence genes such as invA and spiC were found in strains isolated from reptiles (Bruce et al., 2018). However, it must be noted that having one or more virulence genes does not confer pathogenicity on a strain unless the strain acquires the appropriate virulence gene combination to cause disease in a particular host species (Whiley et al., 2017; Xia et al., 2020; Meletiadis et al., 2022).

In the authors’ opinion, the risk of RAS should be effectively communicated, and risk reduction can be most efficiently achieved with public education and the improvement of animal health. Despite the existence of numerous case reports, reviews, and studies on RAS, the risk of developing RAS does not seem to be fully understood within the population of reptile owners as poorly informed owners and inadequate hygiene standards are still observed (Corrente et al., 2017). A recent evaluation of online platforms selling turtles in the USA revealed that turtles, including small turtles <4 inches in diameter, which are banned from sale in many US states, appear to be readily available for purchase. These websites also did not provide adequate information about *Salmonella* or federal regulations (Montague et al., 2022). Therefore, further education efforts are necessary to address this gap in knowledge. The responsibility for establishing and implementing prevention strategies for RAS falls not only on the reptile owners but also on veterinarians and regulatory bodies to ensure that adequate information and guidelines are accessible and widely disseminated.

### 4.3 Prevention and treatment

Considering the well-documented risk of RAS, preventive measures should be implemented to mitigate the zoonotic risk of *Salmonella* in reptiles. Various options have been reported over the decades, and their implementation should prioritize both animal and owner welfare. While restrictions on the trade and keeping of reptile may have a measurable effect on RAS prevalence, such measures also carry the risk of stimulating black-market trading, compromising control, and, even worse, impacting overall animal health by limiting access to veterinary care and equipment for husbandry as well as reducing exchange of information on high quality husbandry (Harris et al., 2010; Montague et al., 2022). Moreover, targeted measures focusing on individual species or reptile groups do not appear to be promising, given the high shedding rates reported across captive reptile groups and species. Implementing such regulations also raises legal concerns in many countries. As pointed out by Pasmans et al. (Pasmans et al., 2017), the risks posed by reptiles as pets should be seen in context, with higher risks for infection or injury in general posed by more traditional pets, and higher risks for human salmonellosis specifically posed e.g., through food.

Numerous approaches have been investigated and implemented to mitigate the occurrence of RAS in the human population. These strategies encompass a wide range of interventions, including the prohibition of keeping certain reptile groups in captivity, public education campaigns, and various methods for assessing and reducing *Salmonella* contamination in reptiles.

#### 4.3.1 Education

Educational initiatives have been identified as crucial in the prevention of RAS (De Jong et al., 2005; Whiley et al., 2017; Meletiadis et al., 2022). Recent studies have shed light on behaviours and knowledge gaps among reptile owners. For instance, research by Meletiadis et al. (2022) revealed that some owners allow their reptiles access to the kitchen and/or bathroom, and handwashing hygiene after handling these animals was found to be suboptimal, with only 69% of owners consistently washing their hands after contact. Notably, in one study only a single family out of 13 with children affected by RAS was aware of the risk before the illness occurred (Colomb-Cotinat et al., 2014). Another study revealed similarly low numbers with 14% of affected RAS patients aware that reptiles may carry *Salmonella* spp. (Bosch et al., 2016). Furthermore, while some owners claimed to be aware of potential risks, they were unable to name any specific hazard (Corrente et al., 2017). However, higher levels of risk awareness have also been reported, with approximately 50%–60% of patients with RAS confirming awareness of the potential risk of RAS in one study (Whitten et al., 2015). Awareness of RAS may also depend on the species owned, as in one study owners of lizards and snakes were reported to have a higher awareness of the risk of RAS compared to turtle owners (Waltenburg et al., 2022). Studies have documented the effectiveness of educational programs targeting reptile owners in reducing numbers of RAS cases (De Jong et al., 2005).

Various approaches can be employed to reach different audiences and contribute to the prevention of RAS. Educational materials should encompass a range of essential information, including hygiene measures such as appropriate hand washing routines. It is crucial to prevent reptiles from freely roaming in rooms inhabited by people and to prohibit their presence in areas such as kitchens, bathrooms, or food preparation areas (Bradley et al., 2001; Warwick et al., 2001).

#### 4.3.2 Reptile bans

In the United States, the implementation of a ban on small turtles with shell diameters smaller than 4 inches in 1975 is estimated to have averted approximately 100.000 cases of RAS annually (Vora et al., 2012). However, the impacts of subsequent regulations within the USA, for example, in relation to childcare centers, have been found to be highly dependent on the respective state (Vora et al., 2012). In Sweden, import restrictions on reptiles were enforced from 1970 until 1994. These regulations prohibited the trade of turtles with shell diameters less than 10 cm and required reptiles to be certified as *Salmonella*-free prior to importation. The effectiveness of these measures in preventing RAS was generally acknowledged (De Jong et al., 2005). The removal of these importation restrictions in 1994 resulted in an initial rise in RAS cases in the country by up to 4.5%–11.6% in the following years. Subsequently, an education program was launched in 1997, which was associated with a significant reduction in RAS cases (De Jong et al., 2005).

#### 4.3.3 Antibiotic treatments

Since reptiles have been shown to be able to carry multi-drug resistant *Salmonella* spp. (D’Aoust et al., 1990; Marin et al., 2020; Marin et al., 2022), treatment can generally be challenging. Treatment of healthy animals is not recommended (Bradley et al., 2001; Pasmans et al., 2008; Romero, 2016) and is unlikely to lead to permanently *Salmonella*-free animals (Mitchell 2011; Pasmans et al., 2008; Romero 2016). Mitchell (2011) recommended removal instead of treatment for *Salmonella*-positive tested animals used for educational programs. Depending on the clinical case, the use of different antibiotic agents has been described in reptiles for the treatment of salmonellosis. Antibiotics frequently reported in the treatment of salmonellosis in reptiles include ceftazidime (Schroff et al., 2010; Barboza et al., 2018), enrofloxacin (Ramsay et al., 2002; Barboza et al., 2018), and amikacin (Clancy et al., 2016a), as well as ampicillin or piperacillin (Ramsay et al., 2002; Barboza et al., 2018). Antibiotic agents should generally be chosen according to microbial susceptibility results (Clancy et al., 2016a).

A study by Marin et al., 2022 reported antibiotic resistance in all the *Salmonella* strains of reptile origin tested, 72% of which were multidrug-resistant strains. The most frequently observed resistance pattern was the combination of gentamicin-colistin and gentamicin-colistin-ampicillin. The high resistance against gentamicin is suspected to be a result of the indiscriminate use of aminoglycosides by pet reptile breeders. Gentamicin is commonly used in the USA as a prophylactic *Salmonella* treatment in eggs to reduce bacterial loads (Siebeling et al., 1984; Mitchell et al., 2007; Marin et al., 2022). Some studies have focused exclusively on the production of *Salmonella*-free reptiles, and various treatment methods have been evaluated, including antimicrobials such as oxytetracycline, gentamicin or chloramphenicol in water or directly on the eggs (Mitchell et al., 2007). However, although some of these methods resulted in the reduction of infection and/or shedding rates in hatchling turtles, their excessive use also increased antibiotic resistance (Mitchell et al., 2007). Other substances, such as sodium hypochlorite and polyhexamethylene biguanides (Mitchell et al., 2007) or chlorhexidine gluconate (Cota et al., 2021) also lead to some degree of reduction in *Salmonella* carriage of hatchlings. However, in general, efforts to eliminate *Salmonella* spp. in reptiles have been unsuccessful (Pasmans et al., 2021).

Not only has the efficacy of antibiotic eradication shown limited success, but the utilization of certain antimicrobial agents, such as 3rd and 4th generation cephalosporins, fluoroquinolones, and other antimicrobials classified by the World Health Organization (WHO) as “Highest Priority Critically Important Antimicrobials (HPCIA),” in veterinary medicine has been subject to critical discussion. This concern is further compounded by the escalating issue of antimicrobial resistance. Consequently, antibiotic treatment for clinically ill animals should rely on guidelines, such as the consensus statement (Weese et al., 2015) from the American College of Veterinary Internal Medicine (ACVIM). Recommendations on the use of antimicrobials in exotic animals, including reptiles, have also been published (Broens and Van Geijlswijk, 2018; Perry and Mitchell, 2019; Hedley et al., 2021; Divers and Burgess, 2023).

#### 4.3.4 Use of phages or probiotics

Bacteriophages have demonstrated effectiveness in moderating bacterial populations in numerous veterinary settings (Filho et al., 2007; Hong, 2013; Cortés et al., 2015; Fadlallah et al., 2015; Wernicki et al., 2017; Gigante and Atterbury, 2019). Several studies have investigated the potential of bacteriophages to reduce *Salmonella* shedding in different animal species, demonstrating some efficacy (Berchieri et al., 1991; Filho et al., 2007; Lim et al., 2012; Adhikari et al., 2017). However, very few studies are currently available describing the use of bacteriophages for the reduction of *Salmonella* in the gut of reptiles. One study demonstrated the ability of a specific phage to replicate in *Salmonella* spp. in the intestines of bearded dragons and thereby to decrease the number of *Salmonella* shed in the feces (Renfert et al., 2021). Another study successfully used a phage to reduce the numbers of *Salmonella* spp. associated with reptile skin (Kwon et al., 2021).

Probiotics have also been suggested to influence reptile health and gut microbiota. Positive effects have been reported, e.g., in turtles, probiotics were associated with an increase in body weight and enhanced shell mineralization (Rawski et al., 2016). However, existing studies on the use of pro- or prebiotics to decrease the shedding of *Salmonella* spp. have not been successful (Holz and Middleton, 2002; 2005).

#### 4.3.5 Optimization of reptile health

Another important measure that should be emphasized to reduce the risk of RAS is the improvement of overall reptile health. Striving to recreate natural conditions is not only a fundamental goal for herpetologists but also a key factor in promoting healthy reptiles and supporting a functional immune system (Pasmans et al., 2008). Welfare assessment methods have been described, with a focus on behavioural criteria being particularly relevant (Warwick et al., 2013; Bashaw et al., 2016; Moszuti et al., 2017; Benn et al., 2019; Whittaker et al., 2021). Although the connection between behavioural enrichment, the immune system, and *Salmonella* has not yet been studied, multiple studies have indicated a relationship between stress, disease, and shedding of *Salmonella* in reptiles (Sting et al., 2013; Clancy et al., 2016b; Bertolini et al., 2021).

### 4.4 Conclusion: a need for further research and education

The data summarized in this review support the assessment of *Salmonella* in reptiles as a common finding and a zoonotic risk, but also illustrate the limited knowledge of physiologic mechanisms and pathogenic processes. Although the reviews were able to summarize and interpret important data, there are still many aspects of *Salmonella* in reptiles that require further study. Exact mechanisms of shedding, interaction, and invasiveness of *Salmonella* are still not well understood. Little is also known about the specific virulence of reptile-associated serovars in humans. Studies investigating the cytotoxicity of *Salmonella* serovars isolated from reptiles have provided initial insights into their pathogenic potential. However, further research is necessary before definitive conclusions can be drawn and comprehensive risk assessments can be conducted.

Therefore, there is a need to initiate further research on

• The role of *Salmonella* in the normal gut microbiome of reptiles
• Factors influencing invasion and pathogenicity of *Salmonella* in reptiles
• Factors influencing and methods for reducing shedding of *Salmonella* in reptiles
• The role of specific strains and their genomic makeup in epidemiology, shedding, and pathogenicity of *Salmonella* for reptiles and humans
• Understanding the role of humans in increasing *Salmonella* prevalence in reptiles

To control the zoonotic risk of *Salmonella* in reptiles, it is essential to

• Improve the general health status of reptiles by providing a physiologic, healthy environment
• Improve hygienic standards for reptiles kept in family homes
• Stimulate education programs focusing on the understanding of needs and risks when keeping exotic animals

## Supplementary material

SUPPLEMENTARY DATA SHEET S1 Prevalence of Salmonella in healthy reptiles survey (2002–2023). Prisma Chart flow.

SUPPLEMENTARY DATA SHEET S2 Prevalence of Salmonella in healthy reptiles survey (2002–2023). Data evaluation details, references see list in Supplementary Data Sheet S3.

SUPPLEMENTARY DATA SHEET S3 Prevalence of Salmonella in healthy reptiles survey (2002–2023). References.

SUPPLEMENTARY DATA SHEET S4 Reptile associated Salmonellosis Survey (2002–2023). Prisma Chart flow.

SUPPLEMENTARY DATA SHEET S5 Reptile associated Salmonllosis Survey (2002–2023). Data evaluation details, references see list in Supplementary Data Sheet S6.

SUPPLEMENTARY DATA SHEET S6 Reptile associated Salmonellosis Survey (2002–2023). References.
