# Supplementary material for: Salmonella in reptiles: a review of occurrence, interactions, shedding and risk factors for human infections
Source: Front Cell Dev Biol. 2023 Sep 26;11:1251036. doi: 10.3389/fcell.2023.1251036 (PMC10562597; doi:10.3389/fcell.2023.1251036)
Supplement: Supplementary file 1 [file DataSheet2.PDF]

Publ. No.	Author and year	Animal Group	Number of individual animals	wild/captive	Sample material (cloaca, faeces, organ…)	cloaca	faeces	other	method serology	method molecular	method unclear	characterzation serovar level yes	characterzation serovar level partially	characterzation serovar level no	I	II	IIIa	IIIb	IV	V	animals positive	detection rate	Location of study (country)	continent
1	Seepersadsingh 2003	reptile nonspec.	14	wild	cloaca	x			x			x									2	14.3	Trinidad	South America
2	Corrente et al. 2004	snake	24	captive	cloaca, faeces	x	x		x				x		x		x				20	83.3		Europe
2	Corrente et al. 2004	lizard	32	captive	cloaca, faeces	x	x		x				x		x		x				19	59.4		Europe
2	Corrente et al. 2004	turtle	29	captive	cloaca, faeces	x	x		x				x		x		x				3	10.3		Europe
3	Richards et al. 2004	turtle	67	wild	cloaca	x					x										0	0.0	USA, Virginia	North America
3	Richards et al. 2004	snake	8	wild	cloaca	x					x										0	0.0		North America
4	Schröter et al. 2004	snake	16	captive	faeces		x		x	x		x						x			13	81.3	Germany	Europe
5	Strohl et al. 2004	tortoise	47	captive	faeces		x		x			x			x	x					44	93.6	France	Europe
5	Strohl et al. 2004	turtle	5	captive	faeces		x				x										0	0.0		Europe
6	Nakadai et al. 2005	snake	23	captive	faeces		x		x				x		x	x	x	x			23	100.0	Japan	Asia
6	Nakadai et al. 2005	lizard	71	captive	faeces		x		x				x		x	x		x	x		47	66.2		Asia
6	Nakadai et al. 2005	tortoise	4	captive	faeces		x		x				x		x	x		x			3	75.0		Asia
6	Nakadai et al. 2005	turtle	14	captive	faeces		x		x				x		x						10	71.4		Asia
7	Chambers et al. 2006	snake	54	wild	cloaca	x			x			x			x						51	94.4	USA, PA	North America
7	Chambers et al. 2006	turtle	10	wild	cloaca	x			x			x			x						10	100.0		North America
8	Gartrell et al. 2006	tuatara	30	captive	cloaca	x					x										0	0.0	New Zealand	Oceania
9	Grupka et al. 2006	snake	36	captive	faeces		x		x			x			x		x	x			36	100.0	USA, TN	North America
10	Saelinger et al. 2006	turtle	94	wild	cloaca, faeces, gastrointestingal mucosa in necropsied animals	x	x	x			x										0	0.0	USA, NC	North America
11	Schröter et al. 2006	snake	20	captive	faeces		x		x			x			x			x			13	65.0	Germany	Europe
12	Bemis et al. 2007	snake	42	captive	faeces		x		x	x		x			x		x	x			17	40.5	USA, TN	North America
13	Gartrell et al. 2007	tuatara	100	wild	cloaca	x					x										0	0.0	New Zealand	Oceania
14	Gaertner et al. 2008	turtle	52	wild	carapace and cloaca	x		x	x	x		x			x						24	46.2	USA, TX	North America
15	Hidalgo-Vila et al. 2007	tortoise	16	wild	cloaca	x			x				x		x	x					16	100.0	Spain	Europe
15	Hidalgo-Vila et al. 2007	turtle	76	wild	cloaca	x			x				x		x	x					10	13.2		Europe
16	Hidalgo-Vila et al. 2008	turtle	172	wild	cloaca	x			x			x			x	x					11	6.4	Spain	Europe
16	Hidalgo-Vila et al. 2008	turtle	39	captive	cloaca	x			x			x			x	x					2	5.1	Spain	Europe
17	Hidalgo-Vila et al. 2008b	tortoise	28	wild	cloaca	x			x			x			x	x					26	92.9	Morocco	Africa
18	Jang et al. 2008	turtle	11	captive	cloaca	x			x												0	0.0	Korea	Asia
18	Jang et al. 2008	lizard	8	captive	cloaca	x			x			x			x						8	100.0		Asia
18	Jang et al. 2008	snake	27	captive	cloaca	x			x			x			x						22	81.5		Asia
19	Lockhart et al. 2008	tortoise	80	wild	cloaca	x			x			x			x						4	5.0	USA, GA	North America
20	Chen et al. 2010	snake	33	captive	cloaca or faeces	x	x		x			x			x						22	66.7	Taiwan	Asia

20	Chen et al. 2010	lizard	43	captive	cloaca or faeces	x	x		x			x			x	x					27	62.8		Asia
20	Chen et al. 2010	turtle	400	captive	cloaca or faeces	x	x		x			x			x						97	24.3		Asia
21	Maciel et al. 2010	lizard	30	captive	cloaca	x			x			x			x						30	100.0	Brazil	South America
22	Parsons 2010	turtle	64	wild	cloaca	x				x											0	0.0	Australia	Oceania
22	Parsons 2010	lizard	298	wild	cloaca	x				x				x	x			x	x		32	10.7		Oceania
22	Parsons 2010	crocodilian	33	wild	cloaca	x				x				x							1	3.0		Oceania
22	Parsons 2010	snake	48	wild	cloaca	x				x											0	0.0		Oceania
23	Callaway et al. 2011	lizard	100	wild	intestinal content			x	x				x		x						7	7.0	Australia	Oceania
24	Franco et al. 2011	lizard	63	wild	cloaca	x			x	x		x			x	x		x	x		62	98.4	Galapagos	South America
25	Kikillus 2011	lizard	115	captive	cloaca	x			x			x			x	x					32	27.8	New Zealand	Oceania
25	Kikillus 2011	turtle	230	captive	cloaca	x			x			x			x						10	4.3		Oceania
25	Kikillus 2011	tortoise	33	captive	cloaca	x			x			x			x						1	3.0		Oceania
26	Percipalle	tortoise	287	captive	cloaca	x			x			x			x	x		x			102	35.5	Italy	Europe
27	Sánchez-Jiménez et al. 2011	turtle	110	captive	cloaca	x				x			x		x						30	27.3	Colombia	South America
28	Scheelings et al. 2011	snake	66	captive	cloaca or intestinal swabs	x		x	x			x			x			x			57	86.4	Australia	Oceania
28	Scheelings et al. 2011	lizard	103	captive	cloaca or intestinal swabs	x		x	x			x			x			x	x		39	37.9		Oceania
28	Scheelings et al. 2011	tortoise	19	captive	cloaca or intestinal swabs	x		x	x			x			x						2	10.5		Oceania
28	Scheelings et al. 2011	turtle	21	captive	cloaca or intestinal swabs	x		x	x												0	0.0		Oceania
28	Scheelings et al. 2011	snake	38	wild	cloaca or intestinal swabs	x		x	x			x			x	x	x	x	x		15	39.5		Oceania
28	Scheelings et al. 2011	lizard	196	wild	cloaca or intestinal swabs	x		x	x			x			x	x		x	x		26	13.3		Oceania
28	Scheelings et al. 2011	tortoise	43	wild	cloaca or intestinal swabs	x		x	x												0	0.0		Oceania
28	Scheelings et al. 2011	turtle	17	wild	cloaca or intestinal swabs	x		x	x												0	0.0		Oceania
29	Uhart 2011	crocodilian	102	captive	cloaca	x			x				x		x						28	27.5	Argentina	South America
30	Dipineto et al. 2012	tortoise	53	captive	cloaca	x			x			x			x	x					26	49.1	Italy	Europe
31	Goupil et al. 2012	snake	12	captive	faeces or cloaca	x	x		x			x			x		x				11	91.7	USA	North America
32	Hydeskov 2012 U(epub) /2013	snake	53	captive	cloaca	x			x			x			x		x	x			33	62.3	Denmark	Europe
32	Hydeskov 2012 U(epub) /2013	reptile nonspec.	66	captive	cloaca	x			x			x			x			x			24	36.4		Europe
32	Hydeskov 2012 U(epub) /2013	lizard	81	captive	cloaca	x			x			x			x				x		12	14.8		Europe
33	Lankau et al. 2012	lizard	79	wild	faeces		x		x	x			x		x	x			x		47	59.5	Galapagos	South America
34	Prapasarakul et al. 2012	snake	128	captive	faeces		x		x	x		x			x			x			128	100.0	Tailand	Asia
35	Smith et al. 2012	lizard	110	wild	faeces		x		x			x			x		x		x		41	37.3	Indonesia	Asia

36	Wheeler et al. 2012	lizard	79	wild	feaces		x		x					x							45	57.0	Galapagos	South America
36	Wheeler et al. 2012	tortoise	10	wild	faeces		x				x										0	0.0		South America
37	Krautwald-Junghanns et al. 2013	snake	85	wild	cloaca	x			x			x			x			x			6	7.1	Germany	Europe
38	Kuroki et al. 2013	snake	87	wild	intestinal contents			x	x			x			x		x	x	x		46	52.9	Japan	Asia
39	Marin 2013	turtle	200	wild	cloaca, water, and intestinal content	x		x	x			x			x	x		x	x		28	14.0	Spain	Europe
40	Martínez et al. 2011	lizard	25	captive	faeces		x		x	x		x			x	x					7	28.0	Spain	Europe
41	Gay et al. 2014	crocodilian	21		cloaca	x			x			x									3	14.3	French Guiana	South America
41	Gay et al. 2014	snake	37		cloaca	x			x			x									12	32.4		South America
41	Gay et al. 2014	lizard	61		cloaca	x			x			x									14	23.0		South America
41	Gay et al. 2014	turtle	32		cloaca	x			x			x									6	18.8		South America
42	Gong et al. 2014	turtle	41	wild	cloaca	x			x			x			x						16	39.0	China	Asia
43	Middleton et al. 2014	tuatara	500	wild	cloaca	x			x												0	0.0	New Zealand	Oceania
43	Middleton et al. 2014	snake	275	wild	cloaca	x			x			x			x						18	6.5		Oceania
44	Schmidt et al. 2014	snake	56	wild	cloaca	x			x			x						x			8	14.3	Germany	Europe
45	Sumiyama et al. 2014	lizard	141	wild	cloaca	x			x			x			x						46	32.6	Japan	Asia
46	Sylvester et al. 2014	lizard	62		cloaca	x			x			x			x						34	54.8	Grenada	South America
47	Wikström et al. 2014	reptile nonspec.	53	captive	cloaca or feaces	x	x		x			x			x	x		x			31	58.5	Sweden	Europe
48	LUKAC 2015	snake	90	captive	Skin, pharyngeal, cloaca and faeces	x	x	x	x				x				x	x			8	8.9	Croatia	Europe
48	LUKAC 2015	lizard	31	captive	Skin, pharyngeal, cloaca and faeces	x	x	x	x			x			x	x		x	x		15	48.4		Europe
48	LUKAC 2015	reptile nonspec.	79	captive	Skin, pharyngeal, cloaca and faeces	x	x	x	x			x			x						3	3.8		Europe
49	Jiménez et al. 2015	lizard	115	wild	gut contents			x	x			x			x						5	4.3	Costa Rica	North America
50	Molina-López et al. 2015	turtle	44	captive	cloaca	x					x	x			x						2	4.5	Spain	Europe
51	Nowakiewicz et al. 2015	turtle	130	wild	cloaca	x			x			x			x						4	3.1	Poland	Europe
52	Bošnjak et al. 2016	snake	34	captive	cloaca and faeces	x	x		x			x			x						5	14.7	Serbia	Europe
52	Bošnjak et al. 2016	lizard	47	captive	cloaca and faeces	x	x		x			x			x			x			10	21.3		Europe
52	Bošnjak et al. 2016	turtle	7	captive	cloaca and faeces	x	x		x			x			x						4	57.1		Europe
53	Corrente et al. 2017	snake	48	captive	cloaca	x				x				x							26	54.2	Italy	Europe
53	Corrente et al. 2017	lizard	42	captive	cloaca	x				x				x							28	66.7		Europe
53	Corrente et al. 2017	turtle	10	captive	cloaca	x				x				x							3	30.0		Europe
54	Ives et al. 2017	turtle	32	wild	cloaca	x			x	x		x			x						2	6.3	Caribbean	North America
55	Bruce et al. 2018	tortoise	89	captive	cloaca	x			x				x		x						5	5.6	UK	Europe

56	Nguyen 2018	lizard	101	wild	faeces		x		x			x			x			x	x		24	23.8	Vietnam	Asia
57	Prud'homme et al. 2018	lizard	277		faeces		x				x	x			x				x		12	4.3	Grand Cayman	North America
57	Prud'homme et al. 2018	lizard	57		faeces		x				x	x			x				x		2	3.5	Grand Cayman	North America
58	Russo et al. 2018	lizard	70	captive	faeces		x		x			x			x			x	x		24	34.3	Italy	Europe
59	Guyomard-Rabenirina et al. 2019	lizard	426		cloaca (anoles), faeces (iguanas and geckos)	x	x		x			x			x				x		64	15.0	Guadeloupe	North America
60	Kuroki 2019	turtle	227	captive	intestine, liver, yolk sac			x	x	x		x			x						190	83.7	Japan	Asia
61	Pulford et al. 2019	snake	106	captive	faeaces		x			x		x			x		x	x			97	91.5	captive: UK; wild: various African countries	Europe
62	Abrahão et al. 2020	lizard	153	wild	cloaca	x			x	x		x			x						67	43.8	Brazil	South America
63	Bjelland 2020	snake	53	captive	cloaca	x			x			x			x	x	x	x			33	62.3	Norway	Europe
63	Bjelland 2020	lizard	15	captive	cloaca	x			x			x			x	x		x	x		10	66.7		Europe
63	Bjelland 2020	turtle	27	captive	cloaca	x			x												0	0.0		Europe
63	Bjelland 2020	tortoise	8	captive	cloaca	x			x			x			x						1	12.5		Europe
64	Rush et al. 2020	snake	45	wild	cloaca	x			x			x			x			x	x		16	35.6	Grenada	North America
65	Sumiyama 2020a	lizard	79	wild	large intestine				x			x			x						24	30.4	Japan	Asia
66	Sumiyama 2020b	lizard	706	wild	large intestine			x	x			x			x						15	2.1	Japan	Asia
67	Cota 2021	snake	8	captive	cloaca	x					x			x			x				4	50.0	Portugal	Europe
67	Cota 2021	lizard	27	captive	cloaca	x					x			x			x				14	51.9		Europe
67	Cota 2021	reptile nonspec.	43	captive	cloaca	x					x			x			x				8	18.6		Europe
68	Baling & Mitchell 2021	lizard	221	wild	cloaca, faeces	x	x		x			x			x						12	5.4	New Zealand	Oceania
69	Calle et al. 2021	turtle	50	captive	cloaca	x					x										0	0.0	Myanmar	Asia
70	Cummings et al. 2021	reptile nonspec.	17	wild	cloaca	x			x			x									1	5.9	USA, NY	North America
71	Doden 2021	turtle	341	wild	cloaca	x			x			x			x						9	2.6	USA, IL, TN	North America
72	Nguyen et al. 2021	lizard	1318	wild	faeces		x		x			x			x			x		x	293	22.2	Cambodia, Thailand, Vietnam	Asia
73	McWhorter et al. 2021	turtle	5	wild	cloaca	x			x			x									1	20.0	Australia	Oceania
73	McWhorter et al. 2021	snake	14	wild	cloaca	x			x			x			x	x			x		7	50.0		Oceania
73	McWhorter et al. 2021	lizard	111	wild	cloaca	x			x			x			x						44	39.6		Oceania
73	McWhorter et al. 2021	tortoise	40	captive	cloaca	x			x			x						x			2	5.0		Oceania
73	McWhorter et al. 2021	turtle	2	captive	cloaca	x			x												0	0.0		Oceania
73	McWhorter et al. 2021	snake	46	captive	cloaca	x			x			x			x			x			26	56.5		Oceania
73	McWhorter et al. 2021	lizard	33	captive	cloaca	x			x			x			x			x			14	42.4		Oceania
73	McWhorter et al. 2021	crocodilian	1	captive	cloaca	x			x												0	0.0		Oceania
74	Zając 2021	snake	358	captive	faeces		x		x	x		x			x	x	x	x	x		327	91.3	Poland	Europe
74	Zając 2021	lizard	276	captive	faeces		x		x	x		x			x	x		x			245	88.8		Europe

74	Zając 2021	tortoise	60	captive	faeces		x		x	x		x			x	x					36	60.0		Europe
74	Zając 2021	crocodilian	2	captive	faeces		x		x	x											0	0.0		Europe
75	Merkevičienė et al. 2022	snake	52	captive	faeces and cloaca	x	x		x						x		x	x	x		41	78.8	Lithuania	Europe
75	Merkevičienė et al. 2022	lizard	21	captive	faeces and cloaca	x	x		x						x						5	23.8		Europe
75	Merkevičienė et al. 2022	tortoise	2	captive	faeces and cloaca	x	x		x												0	0.0		Europe
75	Merkevičienė et al. 2022	snake	17	wild	faeces and cloaca	x	x		x						x						4	23.5		Europe
75	Merkevičienė et al. 2022	lizard	5	wild	faeces and cloaca	x	x		x												0	0.0		Europe
76	Abreu-Acosta et al. 2023	lizard	74	wild	faeces		x			x				x							4	5.4	Tenerife	Africa
77	Song et al. 2023	snake	104	captive	faeces and gastrointestinal mucocal samples		x	x		x				x	x			x	x		32	30.8	China	Asia
77	Song et al. 2023	lizard	21	captive	faeces and gastrointestinal mucocal samples		x	x		x				x	x			x			10	47.6		Asia
77	Song et al. 2023	turtle	52	captive	faeces and gastrointestinal mucocal samples		x	x		x				x	x			x			4	7.7		Asia
