# Supplementary material for: Salmonella in reptiles: a review of occurrence, interactions, shedding and risk factors for human infections
Source: Front Cell Dev Biol. 2023 Sep 26;11:1251036. doi: 10.3389/fcell.2023.1251036 (PMC10562597; doi:10.3389/fcell.2023.1251036)
Supplement: Supplementary file 2 [file DataSheet4.PDF]

Reptile associated Salmonellosis Survey (2003-2023)
Prisma Chart Flow

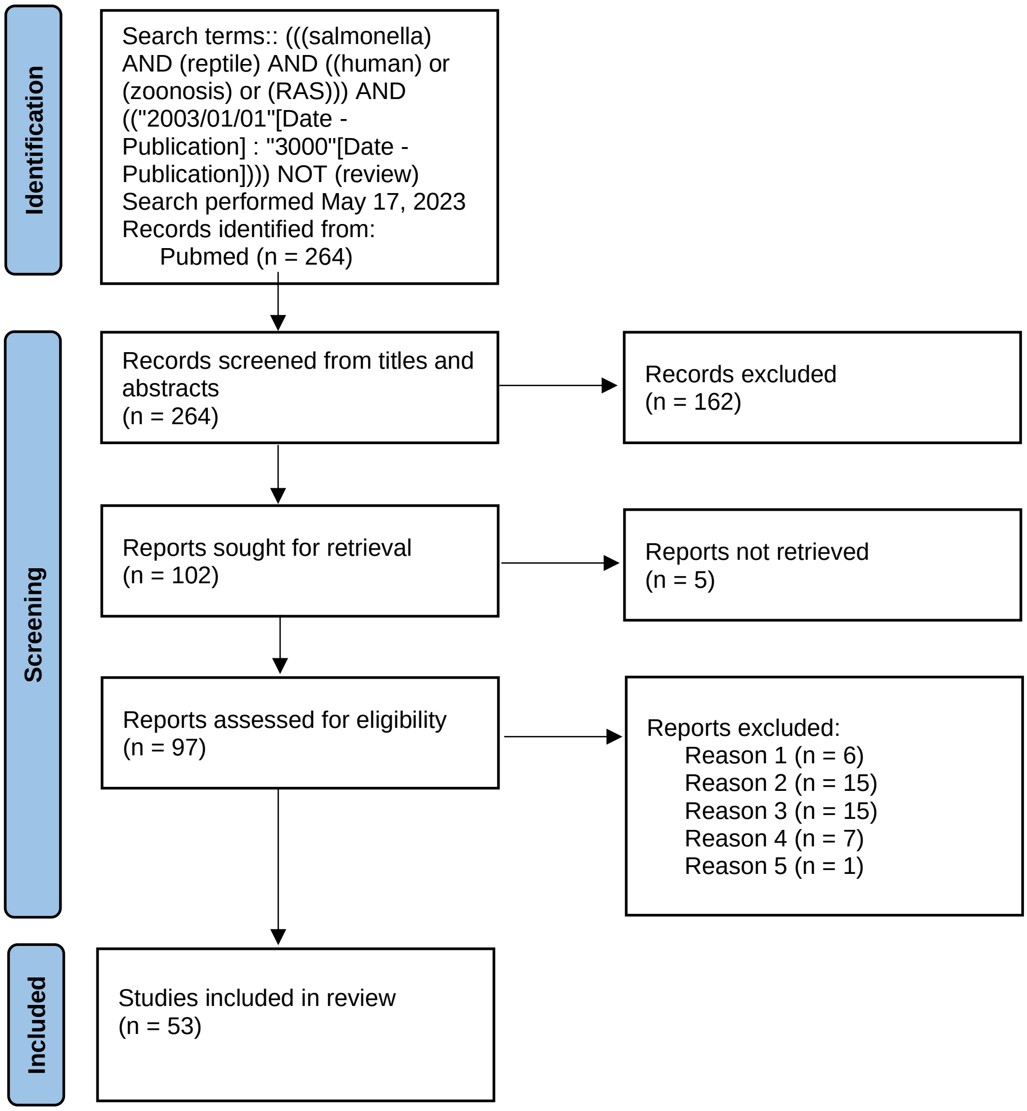

Reasons for exclusion:

1. Lack of information on reptile contact (species of reptile, kind of contact..) (n= 6)
2. Reptiles only mentioned as potential risk but no further investigation (n = 16)
3. No clear association of illness in humans to contact with reptiles (n = 15)
4. Language (Included were: English, German, French) (n = 5)
5. Review itself (n = 1)

Created according to: Page MJ, McKenzie JE, Bossuyt PM, Boutron I, Hoffmann TC, Mulrow CD, et al. The PRISMA 2020 statement: an updated guideline for reporting systematic reviews. BMJ 2021;372:n71. doi: 10.1136/bmj.n7
