# Supplementary material for: Salmonella in reptiles: a review of occurrence, interactions, shedding and risk factors for human infections
Source: Front Cell Dev Biol. 2023 Sep 26;11:1251036. doi: 10.3389/fcell.2023.1251036 (PMC10562597; doi:10.3389/fcell.2023.1251036)
Supplement: Supplementary file 3 [file DataSheet6.pdf]

References Supplement – RAS Table

1. Stam, F., Römkens, T.E., Hekker, T.A., and Smulders, Y.M. (2003). Turtle-associated human salmonellosis. *Clinical Infectious Diseases* 37(11), e167-e169. doi: 10.1086/379612.
2. Mermin, J., Hutwagner, L., Vugia, D., Shallow, S., Daily, P., Bender, J., et al. (2004). Reptiles, amphibians, and human *Salmonella* infection: a population-based, case-control study. *Clinical Infectious Diseases* 38(Supplement_3), S253-S261. doi: 10.1086/381594.
3. Center for Disease Control and Prevention (2005). Salmonellosis associated with pet turtles - Wisconsin and Wyoming, 2004. *MMWR. Morbidity and Mortality Weekly Report* 54(9), 223-226.
4. Foster, N., and Kerr, K. (2005). The snake in the grass - *Salmonella arizonae* gastroenteritis in a reptile handler. *Acta paediatrica (Oslo, Norway: 1992)* 94(8), 1165-1166. doi: 10.1111/j.1651-2227.2005.tb02067.x.
5. Corrente, M., Totaro, M., Martella, V., Campolo, M., Lorusso, A., Ricci, M., et al. (2006). Reptile-associated salmonellosis in man, Italy. *Emerging Infectious Diseases* 12(2), 358-359. doi: 10.3201/eid1202.050692.
6. Kaibu, H., Iida, K., Ueki, S., Ehara, H., Simasaki, Y., Anzai, H., et al. (2006). Salmonellosis of infants presumably originating from an infected turtle in Nagasaki, Japan. *Japanese Journal of Infectious Diseases* 59(4), 281.
7. Milstone, A.M., Agwu, A.G., and Angulo, F.J. (2006). Alerting pregnant women to the risk of reptile-associated salmonellosis. *Obstetrics & Gynecology* 107(2 Part 2), 516-518. doi: 10.1097/01.AOG.0000187950.37065.87.
8. Bélard, S., Kist, M., and Ramharter, M. (2007). Travel-related *Salmonella* Agama, Gabon. *Emerging Infectious Diseases* 13(5), 790.
9. Berendes, T., Keijman, J., Te Velde, L., and Oostenbroek, R. (2007). Splenic abscesses caused by a reptile-associated *Salmonella* infection. *Digestive Surgery* 24(5), 397-399. doi: 10.1159/000107718.
10. Brédart, S., Wastelin, M., Collard, J., Coppée, M., and Bodart, E. (2007). Tortue d'eau et septicémie chez un nourrisson de 4 mois : quel rapport ? *Revue Medicale de Liege* 62(7-8), 496-497.
11. Center for Disease Control and Prevention (2007). Turtle-associated salmonellosis in humans - United States, 2006-2007. *MMWR. Morbidity and Mortality Weekly Report* 56(26), 649-652.
12. Greene, S., Yartel, A., Moriarty, K., Nathan, L., Salehi, E., Tengelsen, L., et al. (2007). *Salmonella* Kingabwa infections and lizard contact, United States, 2005. *Emerging Infectious Diseases* 13(4), 661. doi: 10.3201/eid1304.060888.
13. Bertrand, S., Rimhanen-Finne, R., Weill, F., Rabsch, W., Thornton, L., Perevoščikovs, J., et al. (2008). *Salmonella* infections associated with reptiles: the current situation in Europe. *Eurosurveillance* 13(24), 18902.
14. Fukushima, H., Okuno, J., Fujiwara, Y., Hosoda, T., Kurazono, T., Ohtsuka, K., et al. (2008). An outbreak of *Salmonella* food poisoning at a snapping turtle restaurant. *Journal of the Japanese Association for Infectious Diseases* 61, 328.
15. Hames, A., Mumford, J., Hale, J., and Galloway, A. (2008). *Salmonella* Michigan soft tissue infection in an immunocompromised child. *Journal of Clinical Pathology* 61(6), 773-774. doi: 10.1136/jcp.2007.050468.
16. O'Byrne, A., and Mahon, M. (2008). Reptile-associated salmonellosis in residents in the South East of Ireland 2005 - 2007. *Eurosurveillance* 13(15), 18830. doi: 10.2807/ese.13.15.18830-en.
17. Böhme, H., Fruth, A., and Rabsch, W. (2009). Reptilien-assoziierte Salmonelleninfektionen bei Säuglingen und Kleinkindern in Deutschland. *Klinische Pädiatrie* 221(02), 60-64. doi: 10.1099/jmm.0.012146-0.
18. Böhme, H., Fruth, A., Rebmann, F., Sontheimer, D., and Rabsch, W. (2009). Reptilien-assoziierte Salmonellenenteritis bei einem voll gestillten Säugling. *Klinische Pädiatrie* 221(02), 74-75. doi: 10.1055/s-0028-1112157.
19. Cooke, F.J., De Pinna, E., Maguire, C., Guha, S., Pickard, D.J., Farrington, M., et al. (2009). First report of human infection with *Salmonella enterica* serovar Apapa resulting from exposure to a pet lizard. *Journal of Clinical Microbiology* 47(8), 2672-2674. doi: 10.1128/JCM.02475-08.
20. Harris, J.R., Bergmire-Sweat, D., Schlegel, J.H., Winpisinger, K.A., Klos, R.F., Perry, C., et al. (2009). Multistate outbreak of *Salmonella* infections associated with small turtle exposure, 2007 – 2008. *Pediatrics* 124(5), 1388-1394. doi: 10.1542/peds.2009-0272.
21. Van Meervenne, E., Botteldoorn, N., Lokietek, S., Vatlet, M., Cupa, A., Naranjo, M., et al. (2009). Turtle-associated *Salmonella* septicaemia and meningitis in a 2-month-old baby. *Journal of Medical Microbiology* 58(10), 1379-1381. doi: 10.1099/jmm.0.012146-0.
22. Center for Disease Control and Prevention (2010). Multistate outbreak of human *Salmonella* Typhimurium infections associated with pet turtle exposure-United States, 2008. *MMWR. Morbidity and Mortality Weekly Report* 59(7), 191-196.

23. Moffatt, C.R., Lafferty, A.R., Khan, S., Krsteski, R., Valcanis, M., Powling, J., et al. (2010). *Salmonella* Rubislaw gastroenteritis linked to a pet lizard. *The Medical Journal of Australia* 193(1), 54-55. doi: 10.5694/j.1326-5377.2010.tb03743.x.
24. Tabarani, C.M., Bennett, N.J., Kiska, D.L., Riddell, S.W., Botash, A.S., and Domachowske, J.B. (2010). Empyema of preexisting subdural hemorrhage caused by a rare *Salmonella* species after exposure to bearded dragons in a foster home. *The Journal of Pediatrics* 156(2), 322-323. doi: 10.1016/j.jpeds.2009.07.050.
25. Younus, M., Wilkins, M., Davies, H., Rahbar, M., Funk, J., Nguyen, C., et al. (2010). The role of exposures to animals and other risk factors in sporadic, non-typhoidal *Salmonella* infections in Michigan children. *Zoonoses and Public Health* 57(7-8), e170-e176. doi: 10.1111/j.1863-2378.2010.01324.x.
26. Haase, R., Beier, T., Bernstädt, M., Merkel, N., and Bartnicki, J. (2011). Neugeborenensepsis durch *Salmonella* Apapa nach Reptilienkontakt im Haushalt. *Zeitschrift für Geburtshilfe und Neonatologie* 215(2), 86-88.
27. Lowther, S., Medus, C., Scheftel, J., Leano, F., Jawahir, S., and Smith, K. (2011). Foodborne outbreak of *Salmonella* subspecies IV infections associated with contamination from bearded dragons. *Zoonoses and Public Health* 58(8), 560-566. doi: 10.1111/j.1863-2378.2011.01403.x.
28. Center for Disease Control and Prevention (2012). Notes from the field: outbreak of salmonellosis associated with pet turtle exposures - United States, 2011. *MMWR. Morbidity and Mortality Weekly Report* 61(4), 79.
29. Hernández, E., Rodriguez, J., Herrera-León, S., García, I., De Castro, V., and Muniozguren, N. (2012). *Salmonella* Paratyphi B var Java infections associated with exposure to turtles in Bizkaia, Spain, September 2010 to October 2011. *Eurosurveillance* 17(25).
30. Olariu, A., Jain, S., and Gupta, A.K. (2012). *Salmonella* Kingabwa meningitis in a neonate. *BMJ Case Reports* 2012, 1-2. doi: 10.1136/bcr.10.2011.5032.
31. Lafuente, S., Bellido, J.B., Moraga, F.A., Herrera, S., Yagüe, A., Montalvo, T., et al. (2013). *Salmonella* Paratyphi B and *Salmonella* Litchfield outbreaks associated with pet turtle exposure in Spain. *Enfermedades Infecciosas y Microbiología Clínica* 31(1), 32-35. doi: 10.1016/j.eimc.2012.05.013.
32. Pees, M., Rabsch, W., Plenz, B., Fruth, A., Prager, R., Simon, S., et al. (2013). Evidence for the transmission of *Salmonella* from reptiles to children in Germany, July 2010 to October 2011. *Eurosurveillance* 18(46). doi: 10.2807/1560-7917.es2013.18.46.20634.
33, Basler, C., Bottichio, L., Higa, J., Prado, B., Wong, M., and Bosch, S. (2015). Multistate outbreak of human *Salmonella* Poona infections associated with pet turtle exposure - United States, 2014. *Morbidity and Mortality Weekly Report* 64(29), 804.
34. Murphy, D., and Oshin, F. (2015). Reptile-associated salmonellosis in children aged under 5 years in South West England. *Archives of Disease in Childhood* 100(4), 364-365. doi: 10.1136/archdischild-2014-306134.
35. Bosch, S., Tauxe, R.V., and Behravesh, C.B. (2016). Turtle-associated salmonellosis, United States, 2006–2014. *Emerging Infectious Diseases* 22(7), 1149. doi: 10.3201/eid2207.150685.
36. Gambino-Shirley, K. (2016). Notes from the field: four multistate outbreaks of human *Salmonella* infections linked to small turtle exposure - United States, 2015. *MMWR. Morbidity and Mortality Weekly Report* 65.
37. Angot, M., Labbe, F., Duquenoy, A., and Le Roux, P. (2017). Co-infection rotavirus-*Salmonella* lié aux tortues: à propos de 2 cas de zoonoses domestiques. *Archives de Pédiatrie* 24(8), 747-748. doi: 10.1016/j.arcped.2017.05.001.
38. Baranzelli, A., Loïez, C., Bervar, J., Scherpereel, A., and Wallet, F. (2017). The snake raiser lung: an unusual cause of *Salmonella enterica* subspecies *arizonae* pneumonia. *Médecine et Maladies Infectieuses* 47(6), 424-425. doi: 10.1016/j.medmal.2017.05.001.
39. Draper, A., James, C.L., Pascall, J.E., Shield, K.J., Langrell, J., and Hogg, A. (2017). An outbreak of *Salmonella* Muenchen after consuming sea turtle, Northern Territory, Australia, 2017. 41(4), E290 - E294.
40. Gavrilovici, C., Pânzaru, C.-V., Cozma, S., Mârţu, C., Lupu, V.V., Ignat, A., et al. (2017). "Message from a turtle": otitis with *Salmonella arizonae* in children: case report. *Medicine* 96(44). doi: 10.1097/MD.0000000000008455.
41. Bothwell, R., Sheehy, O., Quinn, M., and Clarke, H. (2018). Do all patients with *Salmonella* infections require a 'pet' scan? *The Ulster Medical Journal* 87(2), 134.
42. Bottichio, L., Webb, L.M., Leos, G., Tolar, B., Dowell, N., and Basler, C. (2018). Notes from the field: *Salmonella* Oranienburg infection linked to consumption of rattlesnake pills - Kansas and Texas, 2017. *Morbidity and Mortality Weekly Report* 67(17), 502-503. doi: 10.15585/mmwr.mm6717a4.
43. Gambino-Shirley, K., Stevenson, L., Concepción-Acevedo, J., Trees, E., Wagner, D., Whitlock, L., et al. (2018). Flea market finds and global exports: four multistate outbreaks of human *Salmonella* infections linked to small turtles, United States - 2015. *Zoonoses and Public Health* 65(5), 560-568. doi: 10.15585/mmwr.mm6525a3.

44. Koski, L. (2018). Notes from the field: an outbreak of *Salmonella* Agbeni infections linked to turtle exposure - United States, 2017. *MMWR. Morbidity and Mortality Weekly Report* 67. doi: 10.15585/mmwr.mm6748a5.
45. Krishnasamy, V., Stevenson, L., Koski, L., Kellis, M., Schroeder, B., Sundararajan, M., et al. (2018). Notes from the field: investigation of an outbreak of *Salmonella* Paratyphi B variant L (+) tartrate+(Java) associated with ball python exposure - United States, 2017. *MMWR. Morbidity and Mortality Weekly Report* 67(19), 562. doi: 10.15585/mmwr.mm6719a7.
46. Koski, L., DeBess, E., Rosen, H.E., Reporter, R., Waltz, T., Leeper, M., et al. (2019). An investigation of *Salmonella* Fluntern illnesses linked to leopard geckos - United States, 2018. *Zoonoses and Public Health* 66(8), 974-977. doi: 10.1111/zph.12647.
47. Kiebler, C.A., Bottichio, L., Simmons, L., Basler, C., Klos, R., Gurfield, N., et al. (2020). Outbreak of human infections with uncommon *Salmonella* serotypes linked to pet bearded dragons, 2012–2014. *Zoonoses and Public Health* 67(4), 425-434. doi: 10.1111/zph.12701.
48. Otake, S., Ajiki, J., Yoshida, M., Koriyama, T., and Kasai, M. (2021). Contact with a snake leading to testicular necrosis due to *Salmonella* Saintpaul infection. *Pediatrics International* 63(1), 119-121. doi: 10.1111/ped.14375.
49. Barry, K., and Finn, A. (2022). *Salmonella* Typhimurium as a causative agent of spontaneous bacterial peritonitis. *BMJ Case Reports CP* 15(4), e249550. doi: 10.1136/bcr-2022-249550.
50. Dewart, C.M., Waltenburg, M.A., Dietrich, S., Machesky, K., Singh, A., Brandt, E., et al. (2022). Cluster of human *Salmonella* Guinea infections: reported reptile exposures and associated opportunities for infection prevention - Ohio, 2019–2020. *Preventive Veterinary Medicine* 198, 105530. doi: 10.1016/j.prevetmed.2021.105530.
51. Meletiadis, A., Biolatti, C., Mugetti, D., Zaccaria, T., Cipriani, R., Pitti, M., et al. (2022). Surveys on exposure to reptile-associated salmonellosis (RAS) in the Piedmont Region - Italy. *Animals* 12(7), 906. doi: 10.3390/ani12070906.
52. Waltenburg, M.A., Perez, A., Salah, Z., Karp, B.E., Whichard, J., Tolar, B., et al. (2022). Multistate reptile-and amphibian-associated salmonellosis outbreaks in humans, United States, 2009 – 2018. *Zoonoses and Public Health* 69(8), 925-937. doi: 10.1111/zph.12990.
53. Bruning, A.H., van den Beld, M., Laverge, J., Welkers, M.R., Kuil, S.D., Bruisten, S.M., et al. (2023). Reptile-associated *Salmonella* urinary tract infection: a case report. *Diagnostic Microbiology and Infectious Disease*, 115889. doi: 10.1016/j.diagmicrobio.2022.115889.
