# Supplementary material for: Salmonella in reptiles: a review of occurrence, interactions, shedding and risk factors for human infections
Source: Front Cell Dev Biol. 2023 Sep 26;11:1251036. doi: 10.3389/fcell.2023.1251036 (PMC10562597; doi:10.3389/fcell.2023.1251036)
Supplement: Supplementary file 4 [file DataSheet3.PDF]

References

1. Seepersadsingh N, Adesiyun AA. Prevalence and antimicrobial resistance of *Salmonella* spp. in pet mammals, reptiles, fish aquarium water, and birds in Trinidad. *J Vet Med B Infect Dis Vet Public Health*. 2003;50(10):488-493. doi:10.1046/j.0931-1793.2003.00710.x
2. Corrente M, Madio A, Friedrich KG, et al. Isolation of *Salmonella* strains from reptile faeces and comparison of different culture media. *J Appl Microbiol*. 2004;96(4):709-715. doi:10.1111/j.1365-2672.2004.02186.x
3. Richards JM, Brown JD, Kelly TR, Fountain AL, Sleeman JM. Absence of detectable *Salmonella* cloacal shedding in free-living reptiles on admission to the wildlife center of Virginia. *J Zoo Wildl Med*. 2004;35(4):562-563. doi:10.1638/03-070
4. Schröter M, Roggentin P, Hofmann J, Speicher A, Laufs R, Mack D. Pet snakes as a reservoir for *Salmonella enterica* subsp. *diarizonae* (Serogroup IIIb): a prospective study. *Appl Environ Microbiol*. 2004;70(1):613-615. doi:10.1128/AEM.70.1.613–615.2004
5. Strohl P, Tilly B, Frémy S, Brisabois A, Guérin-Faublée V. Prevalence of *Salmonella* shedding in faeces by captive chelonians. *Vet Rec*. 2004;154(2):56-58. doi:10.1136/vr.154.2.56
6. Nakadai A, Kuroki T, Kato Y, et al. Prevalence of *Salmonella* spp. in pet reptiles in Japan. *J Vet Med Sci*. 2005;67(1):97-101. doi:10.1292/jvms.67.97
7. Chambers DL, Hulse AC. *Salmonella* serovars in the herpetofauna of Indiana County, Pennsylvania. *Appl Environ Microbiol*. 2006;72(5):3771-3773. doi:10.1128/AEM.72.5.3771-3773.2006
8. Gartrell BD, Jillings E, Adlington BA, Mack H, Nelson NJ. Health screening for a translocation of captive-reared tuatara (*Sphenodon punctatus*) to an island refuge. *N Z Vet J*. 2006;54(6):344-349. doi:10.1080/00480169.2006.36722
9. Grupka LM, Ramsay EC, Bemis DA. *Salmonella* surveillance in a collection of rattlesnakes (*Crotalus* spp.). *J Zoo Wildl Med*. 2006;37(3):306-312. doi:10.1638/05-059.1
10. Saelinger CA, Lewbart GA, Christian LS, Lemons CL. Prevalence of *Salmonella* spp in cloacal, fecal, and gastrointestinal mucosal samples from wild North American turtles. *J Am Vet Med Assoc*. 2006;229(2):266-268. doi:10.2460/javma.229.2.266
11. Schröter M, Speicher A, Hofmann J, Roggentin P. Analysis of the transmission of *Salmonella* spp. through generations of pet snakes. *Environ Microbiol*. 2006;8(3):556-559. doi:10.1111/j.1462-2920.2005.00934.x
12. Bemis DA, Grupka LM, Liamthong S, Folland DW, Sykes JM, Ramsay EC. Clonal relatedness of *Salmonella* isolates associated with invasive infections in captive and wild-caught rattlesnakes. *Vet Microbiol*. 2007;120(3-4):300-307. doi:10.1016/j.vetmic.2006.10.028
13. Gartrell BD, Youl JM, King CM, Bolotovski I, McDonald WL, Nelson NJ. Failure to detect *Salmonella* species in a population of wild tuatara (*Sphenodon punctatus*). *N Z Vet J*. 2007;55(3):134-136. doi:10.1080/00480169.2007.36756
14. Gaertner JP, Hahn D, Rose FL, Forstner MRJ. Detection of salmonellae in different turtle species within a headwater spring ecosystem. *J Wildl Dis*. 2008;44(2):519-526. doi:10.7589/0090-3558-44.2.519
15. Hidalgo-Vila J, Díaz-Paniagua C, Frutos-Escobar C de, Jiménez-Martínez C, Pérez-Santigosa N. *Salmonella* in free living terrestrial and aquatic turtles. *Vet Microbiol*. 2007;119(2-4):311-315. doi:10.1016/j.vetmic.2006.08.012
16. Hidalgo-Vila J, Díaz-Paniagua C, Pérez-Santigosa N, Frutos-Escobar C de, Herrero-Herrero A. *Salmonella* in free-living exotic and native turtles and in pet exotic turtles from SW Spain. *Res Vet Sci*. 2008;85(3):449-452. doi:10.1016/j.rvsc.2008.01.011
17. Hidalgo-Vila J, Díaz-Paniagua C, Ruiz X, et al. *Salmonella* species in free-living spur-thighed tortoises (*Testudo graeca*) in central western Morocco. *Vet Rec*. 2008;162(7):218-219. doi:10.1136/vr.162.7.218

18. Jang YH, Lee SJ, Lim JG, et al. The rate of *Salmonella* spp. infection in zoo animals at Seoul Grand Park, Korea. *J Vet Sci*. 2008;9(2):177-181. doi:10.4142/jvs.2008.9.2.177
19. Lockhart JM, Lee G, Turco J, Chamberlin L. *Salmonella* from gopher tortoises (*Gopherus polyphemus*) in south Georgia. *J Wildl Dis*. 2008;44(4):988-991. doi:10.7589/0090-3558-44.4.988
20. Chen C-Y, Chen W-C, Chin S-C, et al. Prevalence and antimicrobial susceptibility of salmonellae isolates from reptiles in Taiwan. *J Vet Diagn Invest*. 2010;22(1):44-50. doi:10.1177/104063871002200107
21. Maciel BM, Argôlo Filho RC, Nogueira SSC, Dias JCT, Rezende RP. High prevalence of *Salmonella* in tegu lizards (*Tupinambis merianae*), and susceptibility of the serotypes to antibiotics. *Zoonoses Public Health*. 2010;57(7-8):e26-32. doi:10.1111/j.1863-2378.2009.01283.x
22. Parsons SK, Bull CM, Gordon DM. Low prevalence of *Salmonella enterica* in Australian wildlife. *Environ Microbiol Rep*. 2010;2(5):657-659. doi:10.1111/j.1758-2229.2010.00152.x
23. Callaway Z, Thomas A, Melrose W, Buttner P, Speare R. *Salmonella* Virchow and *Salmonella* Weltevreden in a random survey of the Asian house gecko, *Hemidactylus frenatus*, in houses in northern Australia. *Vector Borne Zoonotic Dis*. 2011;11(6):621-625. doi:10.1089/vbz.2010.0015
24. Franco A, Hendriksen RS, Lorenzetti S, et al. Characterization of *Salmonella* occurring at high prevalence in a population of the land iguana *Conolophus subcristatus* in Galápagos Islands, Ecuador. *PLoS One*. 2011;6(8):e23147. doi:10.1371/journal.pone.0023147
25. Kikillus KH, Gartrell BD, Motion E. Prevalence of *Salmonella* spp., and serovars isolated from captive exotic reptiles in New Zealand. *N Z Vet J*. 2011;59(4):174-178. doi:10.1080/00480169.2011.579246
26. Percipalle M, Giardina G, Lipari L, Piraino C, Macrì D, Ferrantelli V. *Salmonella* infection in illegally imported spur-thighed tortoises (*Testudo graeca*). *Zoonoses Public Health*. 2011;58(4):262-269. doi:10.1111/j.1863-2378.2010.01345.x
27. Sánchez-Jiménez MM, Rincón-Ruiz PA, Duque S, et al. *Salmonella enterica* in semi-aquatic turtles in Colombia. *J Infect Dev Ctries*. 2011;5(5):361-364. doi:10.3855/jidc.1126
28. Scheelings TF, Lightfoot D, Holz P. Prevalence of *Salmonella* in Australian reptiles. *J Wildl Dis*. 2011;47(1):1-11. doi:10.7589/0090-3558-47.1.1
29. Uhart M, Ferreyra H, Mattiello R, et al. Isolation of *Salmonella* spp. from yacare caiman (*Caiman yacare*) and broad-snouted caiman (*Caiman latirostris*) from the Argentine Chaco. *J Wildl Dis*. 2011;47(2):271-277. doi:10.7589/0090-3558-47.2.271
30. Dipineto L, Capasso M, Maurelli MP, et al. Survey of co-infection by *Salmonella* and oxyurids in tortoises. *BMC Vet Res*. 2012;8:69. doi:10.1186/1746-6148-8-69
31. Goupil BA, Trent AM, Bender J, Olsen KE, Morningstar BR, Wünschmann A. A longitudinal study of *Salmonella* from snakes used in a public outreach program. *J Zoo Wildl Med*. 2012;43(4):836-841. doi:10.1638/2011-0281R1.1
32. Hydeskov HB, Guardabassi L, Aalbaek B, Olsen KEP, Nielsen SS, Bertelsen MF. *Salmonella* prevalence among reptiles in a zoo education setting. *Zoonoses Public Health*. 2013;60(4):291-295. doi:10.1111/j.1863-2378.2012.01521.x
33. Lankau EW, Cruz Bedon L, Mackie RI. *Salmonella* strains isolated from Galápagos iguanas show spatial structuring of serovar and genomic diversity. *PLoS One*. 2012;7(5):e37302. doi:10.1371/journal.pone.0037302
34. Prapasarakul N, Pulsrikarn C, Vasaruchapong T, et al. *Salmonella* serovar distribution in cobras (*Naja kaouthia*), snake-food species, and farm workers at Queen Saovabha Snake Park, Thailand. *J Vet Diagn Invest*. 2012;24(2):288-294. doi:10.1177/1040638711434110
35. Smith KF, Yabsley MJ, Sanchez S, Casey CL, Behrens MD, Hernandez SM. *Salmonella* isolates from wild-caught Tokay geckos (*Gekko gecko*) imported to the U.S. from Indonesia. *Vector Borne Zoonotic Dis*. 2012;12(7):575-582. doi:10.1089/vbz.2011.0899

36. Wheeler E, Hong P-Y, Bedon LC, Mackie RI. Carriage of antibiotic-resistant enteric bacteria varies among sites in Galapagos reptiles. *J Wildl Dis*. 2012;48(1):56-67. doi:10.7589/0090-3558-48.1.56
37. Krautwald-Junghanns M-E, Stenkat J, Szabo I, et al. Characterization of *Salmonella* isolated from captive and free-living snakes in Germany. *Berl Munch Tierarztl Wochenschr*. 2013;126(5-6):209-215.
38. Kuroki T, Ishihara T, Furukawa I, Okatani AT, Kato Y. Prevalence of *Salmonella* in wild snakes in Japan. *Jpn J Infect Dis*. 2013;66(4):295-298. doi:10.7883/yoken.66.295
39. Marin C, Ingresa-Capaccioni S, González-Bodi S, Marco-Jiménez F, Vega S. Free-living turtles are a reservoir for *Salmonella* but not for *Campylobacter*. *PLoS One*. 2013;8(8):e72350. doi:10.1371/journal.pone.0072350
40. Martínez R, Sánchez S, Alonso JM, et al. *Salmonella* spp. and Shiga toxin-producing *Escherichia coli* prevalence in an ocellated lizard (*Timon lepidus*) research center in Spain. *Foodborne Pathog Dis*. 2011;8(12):1309-1311. doi:10.1089/fpd.2011.0885
41. Gay N, Le Hello S, Weill F-X, Thoisy B de, Berger F. *Salmonella* serotypes in reptiles and humans, French Guiana. *Vet Microbiol*. 2014;170(1-2):167-171. doi:10.1016/j.vetmic.2014.01.024
42. Gong S, Wang F, Shi H, et al. Highly pathogenic *Salmonella Pomona* was first isolated from the exotic red-eared slider (*Trachemys scripta elegans*) in the wild in China: Implications for public health. *Sci Total Environ*. 2014;468-469:28-30. doi:10.1016/j.scitotenv.2013.08.025
43. Middleton DMRL, La Flamme AC, Gartrell BD, Nelson NJ. Reptile reservoirs and seasonal variation in the environmental presence of *Salmonella* in an island ecosystem, Stephens Island, New Zealand. *J Wildl Dis*. 2014;50(3):655-659. doi:10.7589/2013-10-277
44. Schmidt V, Mock R, Burgkhardt E, et al. Cloacal aerobic bacterial flora and absence of viruses in free-living slow worms (*Anguis fragilis*), grass snakes *(Natrix natrix*) and European Adders (*Vipera berus*) from Germany. *Ecohealth*. 2014;11(4):571-580. doi:10.1007/s10393-014-0947-6
45. Sumiyama D, Izumiya H, Kanazawa T, Murata K. *Salmonella* infection in green anoles (*Anolis carolinensis*), an invasive alien species on Chichi Island of the Ogasawara archipelago in Japan. *J Vet Med Sci*. 2014;76(3):461-465. doi:10.1292/jvms.13-0217
46. Sylvester WRB, Amadi V, Pinckney R, et al. Prevalence, serovars and antimicrobial susceptibility of *Salmonella* spp. from wild and domestic green iguanas (*Iguana iguana*) in Grenada, West Indies. *Zoonoses Public Health*. 2014;61(6):436-441. doi:10.1111/zph.12093
47. Wikström VO, Fernström L-L, Melin L, Boqvist S. *Salmonella* isolated from individual reptiles and environmental samples from terraria in private households in Sweden. *Acta Vet Scand*. 2014;56(1):7. doi:10.1186/1751-0147-56-7
48. Lukac M, Pedersen K, Prukner-Radovcic E. Prevalence of *Salmonella* in captive reptiles from croatia. *J Zoo Wildl Med*. 2015;46(2):234-240. doi:10.1638/2014-0098R1.1
49. Jiménez RR, Barquero-Calvo E, Abarca JG, Porras LP. *Salmonella* Isolates in the Introduced Asian House Gecko (*Hemidactylus frenatus*) with Emphasis on *Salmonella* Weltevreden, in Two Regions in Costa Rica. *Vector Borne Zoonotic Dis*. 2015;15(9):550-555. doi:10.1089/vbz.2015.1785
50. Molina-López RA, Vidal A, Obón E, Martín M, Darwich L. Multidrug-resistant *Salmonella enterica* Serovar Typhimurium Monophasic Variant 4,12:i:- Isolated from Asymptomatic Wildlife in a Catalonian Wildlife Rehabilitation Center, Spain. *J Wildl Dis*. 2015;51(3):759-763. doi:10.7589/2015-01-019
51. Nowakiewicz A, Ziółkowska G, Zięba P, et al. Aerobic bacterial microbiota isolated from the cloaca of the European pond turtle (*Emys orbicularis*) in Poland. *J Wildl Dis*. 2015;51(1):255-259. doi:10.7589/2013-07-157
52. Bošnjak I, Zdravković N, Čolović S, et al. Neglected zoonosis: The prevalence of *Salmonella* spp. in pet reptiles in Serbia. *Vojnosanit Pregl*. 2016;73(10):980-982. doi:10.2298/VSP160809222B

53. Corrente M, Sangiorgio G, Grandolfo E, et al. Risk for zoonotic *Salmonella* transmission from pet reptiles: A survey on knowledge, attitudes and practices of reptile-owners related to reptile husbandry. *Prev Vet Med*. 2017;146:73-78. doi:10.1016/j.prevetmed.2017.07.014
54. Ives A-K, Antaki E, Stewart K, et al. Detection of *Salmonella enterica* Serovar Montevideo and Newport in Free-ranging Sea Turtles and Beach Sand in the Caribbean and Persistence in Sand and Seawater Microcosms. *Zoonoses Public Health*. 2017;64(6):450-459. doi:10.1111/zph.12324
55. Bruce HL, Barrow PA, Rycroft AN. Zoonotic potential of *Salmonella enterica* carried by pet tortoises. *Vet Rec*. 2018;182(5):141. doi:10.1136/vr.104457
56. Nguyen KT, Hasegawa M, Nguyen TT, et al. The importance of wild gecko as a source of human *Salmonella* infection. *J Vet Med Sci*. 2018;80(8):1345-1347. doi:10.1292/jvms.18-0233
57. Prud'homme Y, Burton FJ, McClave C, Calle PP. Prevalence, incidence, and identification of *Salmonella enterica* from wild and captive grand cayman iguanas (*Cyclura lewisi*). *J Zoo Wildl Med*. 2018;49(4):959-966. doi:10.1638/2017-0234.1
58. Russo TP, Varriale L, Borrelli L, et al. *Salmonella* serotypes isolated in geckos kept in seven collections in southern Italy. *J Small Anim Pract*. 2018;59(5):294-297. doi:10.1111/jsap.12808
59. Guyomard-Rabenirina S, Weill F-X, Le Hello S, et al. Reptiles in Guadeloupe (French West Indies) are a reservoir of major human *Salmonella enterica* serovars. *PLoS One*. 2019;14(7):e0220145. doi:10.1371/journal.pone.0220145
60. Kuroki T, Ishihara T, Nakajima N, Furukawa I, Une Y. Prevalence of *Salmonella enterica* Subspecies *enterica* in Red-Eared Sliders *Trachemys scripta elegans* Retailed in Pet Shops in Japan. *Jpn J Infect Dis*. 2019;72(1):38-43. doi:10.7883/yoken.JJID.2018.140
61. Pulford CV, Wenner N, Redway ML, et al. The diversity, evolution and ecology of *Salmonella* in venomous snakes. *PLoS Negl Trop Dis*. 2019;13(6):e0007169. doi:10.1371/journal.pntd.0007169
62. Abrahão CR, Moreno LZ, Silva JCR, et al. *Salmonella enterica* in Invasive Lizard from Fernando de Noronha Archipelago: Serotyping, Antimicrobial Resistance and Molecular Epidemiology. *Microorganisms*. 2020;8(12). doi:10.3390/microorganisms8122017
63. Bjelland AM, Sandvik LM, Skarstein MM, Svendal L, Debenham JJ. Prevalence of *Salmonella* serovars isolated from reptiles in Norwegian zoos. *Acta Vet Scand*. 2020;62(1):3. doi:10.1186/s13028-020-0502-0
64. Rush EM, Amadi VA, Johnson R, Lonce N, Hariharan H. *Salmonella* serovars associated with Grenadian tree boa (*Corallus grenadensis*) and their antimicrobial susceptibility. *Vet Med Sci*. 2020;6(3):565-569. doi:10.1002/vms3.234
65. Sumiyama D, Hayashida I, Kanazawa T, Anzai H, Murata K. Prevalence and antimicrobial-resistance profiles of *Salmonella* spp. isolated from green anoles (*Anolis carolinensis*) collected on the Haha-jima of the Ogasawara archipelago, Japan. *J Vet Med Sci*. 2020;82(10):1558-1561. doi:10.1292/jvms.19-0632
66. Sumiyama D, Shimizu A, Kanazawa T, Anzai H, Murata K. Prevalence of *Salmonella* in green anoles (Anolis *Carolinensis*), an invasive alien species in Naha and Tomigusuku Cities, Okinawa Main Island, Japan. *J Vet Med Sci*. 2020;82(5):678-680. doi:10.1292/jvms.19-0594
67. Cota JB, Carvalho AC, Dias I, Reisinho A, Bernardo F, Oliveira M. *Salmonella* spp. in Pet Reptiles in Portugal: Prevalence and Chlorhexidine Gluconate Antimicrobial Efficacy. *Antibiotics (Basel)*. 2021;10(3). doi:10.3390/antibiotics10030324
68. Baling M, Mitchell C. Prevalence of *Salmonella* spp. in translocated wild reptiles and effect of duration of quarantine on their body condition. *N Z Vet J*. 2021;69(3):174-179. doi:10.1080/00480169.2021.1890647
69. Calle PP, Raphael BL, Lwin T, et al. Burmese roofed turtle (*Batagur trivitata*) disease screening in Myanmar. *J Zoo Wildl Med*. 2021;52(4):1270-1274. doi:10.1638/2021-0017

70. Cummings KJ, Siler JD, Abou-Madi N, et al. *Salmonella* isolated from central new york wildlife admitted to a veterinary medical teaching hospital. *J Wildl Dis*. 2021;57(4):743-748. doi:10.7589/JWD-D-20-00231
71. Doden G, Gartlan B, Klein K, Maddox CW, Adamovicz LA, Allender MC. Prevalence and antimicrobial resistance patterns of *Salmonella* spp. in two free-ranging populations of eastern box turtles (*Terrapene carolina carolina*). *J Zoo Wildl Med*. 2021;52(3):863-871. doi:10.1638/2020-0061
72. Nguyen KT, Hasegawa M, Vo TMT, et al. Wild geckos considered as the natural reservoir of *Salmonella* Weltevreden in Southeast Asian countries. *Zoonoses Public Health*. 2021;68(7):815-822. doi:10.1111/zph.12873
73. McWhorter A, Owens J, Valcanis M, et al. In vitro invasiveness and antimicrobial resistance of *Salmonella enterica* subspecies isolated from wild and captive reptiles. *Zoonoses Public Health*. 2021;68(5):402-412. doi:10.1111/zph.12820
74. Zając M, Skarżyńska M, Lalak A, et al. *Salmonella* in Captive Reptiles and Their Environment-Can We Tame the Dragon? *Microorganisms*. 2021;9(5). doi:10.3390/microorganisms9051012
75. Merkevičienė L, Butrimaitė-Ambrozevičienė Č, Paškevičius G, et al. Serological Variety and Antimicrobial Resistance in *Salmonella* Isolated from Reptiles. *Biology (Basel)*. 2022;11(6). doi:10.3390/biology11060836
76. Abreu-Acosta N, Pino-Vera R, Izquierdo-Rodríguez E, Afonso O, Foronda P. Zoonotic Bacteria in *Anolis* sp., an Invasive Species Introduced to the Canary Islands (Spain). *Animals (Basel)*. 2023;13(3). doi:10.3390/ani13030414
77. Song D, He X, Chi Y, et al. Cytotoxicity and Antimicrobial Resistance of *Salmonella enterica* Subspecies Isolated from Raised Reptiles in Beijing, China. *Animals (Basel)*. 2023;13(2). doi:10.3390/ani13020315
