# Supplementary material for: Salmonella in reptiles: a review of occurrence, interactions, shedding and risk factors for human infections
Source: Front Cell Dev Biol. 2023 Sep 26;11:1251036. doi: 10.3389/fcell.2023.1251036 (PMC10562597; doi:10.3389/fcell.2023.1251036)
Supplement: Supplementary file 6 [file DataSheet5.PDF]

Number of Publication	First Author	Animal Group	reptile source	no. Confirmed human cases	age human <1J	age human <4J	age human >4	gastrointestinal	fever	respiratory	CNS or systemic	other	I	II	IIIa	IIIb	IV	V	method serology	method molecualr	method unclear	characterization to serovar level	transmission direct	transmission indirect	serovar confirmed in reptile	Location of study (country)	continent
1	Stam 2003	reptile nonspec.	pet	1			x	x					x							x		yes			yes	Netherlands	Europe
2	Mermin 2004	reptile nonspec.	unknown	50				x					x								x	no			unknown	USA	North America
3	Centers for Disease Control and Prevention 2005	turtle	pet	6		x	x	x				x	x							x		yes			yes	USA	North America
4	Foster 2005	snake	pet	1			x					x			x						x	no	x		yes	Italy	Europe
5	Corrente 2006	snake	pet	1			x	x					x							x		yes	x		yes	Italy	Europe
6	Kaibu 2006	turtle	pet	2			x	x					x							x		no	x		yes	Japan	Asia
7	Milstone 2006	lizard	pet	1	x				x	x	x		x						x			yes		x	unknown	USA	North America
8	Berendes 2007	lizard	pet	1			x	x	x		x		x								x	yes	x		yes	Netherlands	Europe
9	Brédart 2007	turtle	pet	1	x			x	x		x		x						x			yes			yes	Belgium	Europe
10	Centers for Disease Control and Prevention 2007	turtle	pet	23	x	x	x	x	x		x		x						x	x		yes	x	x	yes	USA	North America
11	Ewen 2007	lizard	unknown	1			x	x	x	x			x								x	yes			no	Gabon	Africa
12	Greene 2007	lizard	pet	6	x		x						x						x	x		yes	x	x	no	USA	North America
13	Bertrand 2008	turtle	pet	1	x						x		x								x	yes			unknown	Belgium	Europe
13	Bertrand 2008	snake	pet	3	x		x								x						x	yes	x		unknown	Belgium	Europe
13	Bertrand 2008	snake	pet	3	x		x						x			x					x	yes			no	Finnland	Europe
13	Bertrand 2008	turtle	pet	6									x								x	yes			yes	Finnland	Europe
13	Bertrand 2008	reptile nonspec.	other	3	x	x	x						x								x	yes	x	x	unknown	France	Europe
13	Bertrand 2008	lizard	pet	3	x		x						x								x	yes			unknown	Ireland	Europe
13	Bertrand 2008	turtle	unknown	5	x		x						x								x	yes			unknown	Ireland	Europe
13	Bertrand 2008	snake	pet	1	x											x					x	yes			unknown	Ireland	Europe
13	Bertrand 2008	reptile nonspec.	unknown	3	x		x						x		x						x	yes			unknown	Ireland	Europe
13	Bertrand 2008	lizard	unknown	16	x	x	x	x	x				x	x			x		x			yes			unknown	Germany	Europe
13	Bertrand 2008	snake	unknown	12	x	x	x						x		x	x	x		x			yes			unknown	Germany	Europe
13	Bertrand 2008	turtle	unknown	1	x								x						x			yes			unknown	Germany	Europe
13	Bertrand 2008	reptile nonspec.	unknown	5	x	x	x						x	x			x		x			yes			unknown	Germany	Europe
14	Fukushima 2008	tortoise	unknown	4	x			x	x				x							x		yes	x		yes	Japan	Asia
15	Hames 2008	tortoise	pet	1			x					x	x						x			yes		x	yes	England	Europe
16	O'Byrne 2008	lizard	pet	1			x	x	x			x	x						x		x	yes	x		no	Ireland	Europe
16	O'Byrne 2008	tortoise	pet	2	x		x	x	x				x						x		x	yes	x	x	no	Ireland	Europe
16	O'Byrne 2008	snake	pet	1	x			x		x						x			x		x	yes		x	no	Ireland	Europe
16	O'Byrne 2008	snake	pet	1	x			x							x				x		x	yes		x	yes	Ireland	Europe
17	Böhme 2009	lizard	pet	1	x			x	x				x						x	x		yes		x	yes	Germany	Europe
18	Böhme 2009	lizard	unknown	14	x	x							x	x			x		x	x		yes			unknown	Germany	Europe
18	Böhme 2009	snake	unknown	7	x	x							x		x	x	x		x	x		yes			unknown	Germany	Europe
18	Böhme 2009	turtle	unknown	1		x							x						x			yes			unknown	Germany	Europe
18	Böhme 2009	reptile nonspec.	unknown	5	x	x							x	x			x		x	x		yes			unknown	Germany	Europe
19	Cooke 2009	lizard	pet	1			x	x	x	x	x		x						x	x		yes		x	yes	Germany	Europe
19	Cooke 2009	lizard		3	x								x						x	x		yes		x	yes	Germany	Europe
20	Harris 2009	turtle	pet	47	x	x	x	x	x			x	x							x		yes			yes	USA	North America
20	Harris 2009	turtle	pet	72				x					x							x		yes			yes	USA	North America
20	Harris 2009	reptile nonspec.	pet	4				x					x							x		yes			unknown	USA	North America
21	Van Meervenne 2009	turtle	pet	1	x						x		x							x		yes			yes	Belgium	Europe
22	Centers for Disease Control and Prevention 2010	turtle	pet	26		x	x	x					x							x		yes			yes	USA	North America
22	Centers for Disease Control and Prevention 2010	reptile nonspec.	pet	7	x	x	x	x					x							x		yes			yes	USA	North America
22	Centers for Disease Control and Prevention 2010	turtle	pet	18	x	x	x	x					x							x		yes			unknown	USA	North America
22	Centers for Disease Control and Prevention 2010	reptile nonspec.	pet	3	x	x	x	x					x							x		yes			unknown	USA	North America
23	Moffat 2010	lizard	pet	1	x			x	x				x								x	yes		x	yes	Australia	Oceania
24	Tabarani 2010	lizard	pet	1	x			x			x						x		x			yes		x	no	USA	North America

24	Tabarani 2010	lizard	pet	1	x			x			x						x		x			yes		x	unknown	USA	North America
25	Younus 2010	reptile nonspec.	unknown	18	x	x	x						x								x	yes			no	USA	North America
26	Haase 2011	lizard	pet	1	x						x		x						x			yes		x	yes	Germany	Europe
27	Lowther 2011	lizard	pet	19			x	x	x			x	x				x		x	x		yes		x	yes	USA	North America
28	Centers for Disease Control and Prevention 2012	turtle	pet	36	x	x	x						x								x	yes			yes	USA	North America
29	Hernandez 2012	turtle	pet	6	x	x	x						x						x			yes			yes	Spain	Europe
30	Olariu 2012	multiple	pet	1	x			x			x		x						x			yes		x	no	England	Europe
31	Lafuente 2013	turtle	pet	5	x	x	x	x	x				x						x			yes	x		yes	Spain	Europe
31	Lafuente 2013	turtle	pet	1	x			x	x				x							x		yes		x	yes	Spain	Europe
32	Pees 2013	lizard	pet	16	x	x		x	x				x	x	x	x	x			x		yes			yes	Germany	Europe
32	Pees 2013	snake	pet	4		x	x	x	x				x		x	x				x		yes			yes	Germany	Europe
32	Pees 2013	turtle	pet	2	x			x	x				x				x			x		yes			yes	Germany	Europe
33	Murphy 2014	reptile nonspec.	unknown	48	x	x	x	x	x		x		x		x						x	yes			unknown	England	Europe
34	Basler 2015	turtle	pet	40	x	x	x						x								x	yes			yes	USA	North America
35	Bosch 2015	turtle	pet	921	x	x	x						x							x		yes			unknown	USA	North America
36	Gambino-Shirley 2016	turtle	pet	50	x	x	x						x							x		yes			yes	USA	North America
37	Angot 2017	turtle	pet	2	x			x			x (hypotension)		x						x			yes		x	unknown	France	Europe
38	Baranzelli 2017	snake	pet	1			x			x									x			partially	x		no	France	Europe
39	Draper 2017	turtle	wild	7		x	x	x	x		x		x								x	yes	x		no	Australia	Oceania
40	Gavrilovici 2017	turtle	wild	1			x					x				x			x			yes		x	no	Romania	Europe
41	Bothwell 2018	snake	pet	1			x				x		x						x		x	no			no	Ireland	Europe
42	Bottichio 2018	snake	unknown	2									x							x		yes		x	yes	USA	North America
43	Gambino-Shirley 2018	turtle	pet	56	x	x	x	x			x		x							x		yes			yes	USA	North America
44	Koski 2018	turtle	pet	23	x	x	x						x							x		yes			unknown	USA	North America
45	Krishnasamy 2018	snake	pet	5	x	x	x						x							x		yes			yes	USA	North America
46	Koski 2019	lizard	pet		x	x	x						x							x		yes	x		yes	USA	North America
47	Kiebler 2020	lizard	pet	96	x	x	x	x			x		x						x			yes	x	x	yes	USA	North America
48	Otake 2020	snake	pet	1			x	x				x	x						x			yes			no	Japan	Asia
49	Barry 2022	lizard	pet	1			x	x					x							x		no	x		unknown	USA	North America
50	Dewart 2022	lizard	pet	5		x	x						x							x		yes	x		unknown	USA	North America
50	Dewart 2022	lizard	unknown	41		x	x						x							x		yes	x		unknown	USA	North America
51	Meletiadis 2022	turtle	other	3	x		x						x							x		yes	x	x	no	Italy	Europe
51	Meletiadis 2022	turtle	pet	5		x	x						x							x		yes	x		yes	Italy	Europe
52	Waltenburg 2022	turtle	pet	1007	x	x	x						x							x		yes			yes	USA	North America
52	Waltenburg 2022	lizard	pet	209	x	x	x						x							x		yes			yes	USA	North America
52	Waltenburg 2022	snake	pet	11	x	x	x						x							x		yes			yes	USA	North America
53	Bruning 2023	snake	pet	1			x					x	x						x	x		yes	x		yes	Netherlands	Europe
